# Metagenomic Insights Into the Cycling of Dimethylsulfoniopropionate and Related Molecules in the Eastern China Marginal Seas

**DOI:** 10.3389/fmicb.2020.00157

**Published:** 2020-02-18

**Authors:** Delei Song, Yunhui Zhang, Ji Liu, Haohui Zhong, Yanfen Zheng, Shun Zhou, Min Yu, Jonathan D. Todd, Xiao-Hua Zhang

**Affiliations:** ^1^MOE Key Laboratory of Marine Genetics and Breeding, College of Marine Life Sciences, Ocean University of China, Qingdao, China; ^2^Laboratory for Marine Ecology and Environmental Science, Qingdao National Laboratory for Marine Science and Technology, Qingdao, China; ^3^School of Biological Sciences, University of East Anglia, Norwich Research Park, Norwich, United Kingdom; ^4^Institute of Evolution and Marine Biodiversity, Ocean University of China, Qingdao, China

**Keywords:** DMSP, DMS, sulfur cycle, metagenome, marine sediment, marginal seas

## Abstract

The microbial cycling of dimethylsulfoniopropionate (DMSP) and its gaseous catabolites dimethylsulfide (DMS) and methanethiol (MeSH) are important processes in the global sulfur cycle, marine microbial food webs, signaling pathways, atmospheric chemistry, and potentially climate regulation. Many functional genes have been identified and used to study the genetic potential of microbes to produce and catabolize these organosulfur compounds in different marine environments. Here, we sampled seawater, marine sediment and hydrothermal sediment, and polymetallic sulfide in the eastern Chinese marginal seas and analyzed their microbial communities for the genetic potential to cycle DMSP, DMS, and MeSH using metagenomics. DMSP was abundant in all sediment samples, but was fivefold less prominent in those from hydrothermal samples. Indeed, Yellow Sea (YS) sediment samples had DMSP concentrations two orders of magnitude higher than in surface water samples. Bacterial genetic potential to synthesize DMSP (mainly in *Rhodobacteraceae* bacteria) was far higher than for phytoplankton in all samples, but particularly in the sediment where no algal DMSP synthesis genes were detected. Thus, we propose bacteria as important DMSP producers in these marine sediments. DMSP catabolic pathways mediated by the DMSP lyase DddP (prominent in *Pseudomonas* and *Mesorhizobium* bacteria) and DMSP demethylase DmdA enzymes (prominent in *Rhodobacteraceae* bacteria) and MddA-mediated MeSH S-methylation were very abundant in Bohai Sea and Yellow Sea sediments (BYSS) samples. In contrast, the genetic potential for DMSP degradation was very low in the hydrothermal sediment samples—*dddP* was the only catabolic gene detected and in only one sample. However, the potential for DMS production from MeSH (*mddA*) and DMS oxidation (*dmoA* and *ddhA*) was relatively abundant. This metagenomics study does not provide conclusive evidence for DMSP cycling; however, it does highlight the potential importance of bacteria in the synthesis and catabolism of DMSP and related compounds in diverse sediment environments.

## Introduction

Approximately eight billion tons of the organosulfur compound dimethylsulfoniopropionate (DMSP) is produced in Earth’s surface oceans (Galí et al., 2015). Many marine phytoplankton, bacteria, corals, and some plants produce DMSP as an anti-stress compound (Zhang et al., 2019). Once released into the environment, DMSP is a key marine nutrient (Curson et al., 2011a; Zhang et al., 2019), a chemoattractant for marine organisms (Seymour et al., 2010), and it is a major precursor for the climate active gases dimethylsulfide (DMS) and methanethiol (MeSH). DMS is the primary marine source of sulfur delivered to the atmosphere (Lovelock et al., 1972), where DMS oxidative products act as cloud condensation nuclei and may affect the climate (Stefels et al., 2007; Vallina and Simó, 2007). DMSP and the trace gases DMS and MeSH are important components of the global sulfur cycle, and metabolic pathways (Yoch, 2002).

**FIGURE 1:**
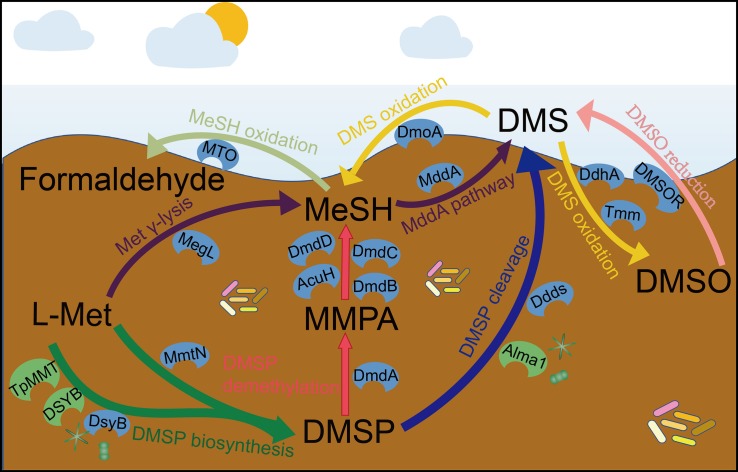
The known steps and key enzymes of each pathway in the production and cycling of DMSP and related compounds. Colored arrows represent individual pathways. The known key enzymes of these pathways are indicated; blue color are largely bacterial whereas those in green are eukaryotic. For DMSP synthesis, the two major pathways are represented by a split arrow. DMS, dimethylsulfide; DMSP, dimethylsulfoniopropionate; DMSO, dimethyl sulfoxide; MMPA, methylmercaptopropionate; MeSH, methanethiol; Ddds, various DMSP lyases; L-Met, L-methionine; Alma1, DMSP lyases; DsyB, DSYB; TpMMT, methylthiohydroxybutryrate SAM-dependent methyltransferase; MmtN, Met-methylating enzymes; MddA, MeSH *S*-methyltransferase; DmoA, dimethylsulfide monooxgenase; MTO, MeSH oxidase; DdhA, dimethylsulfide dehydrogenase; Tmm, trimethylamine monooxygenase; DMSOR, dimethyl sulfoxide reductase; MegL, methionine γ-lyase; DmdA, DMSP demethylase; DmdB, MMPA-CoA ligase; DmdC, MMPA-CoA dehydrogenase; DmdD, methylthioacryloyl-CoA hydratase; AcuH, acryloyl-CoA hydratase.

DMSP is synthesized by three known pathways (Figure 1): the methionine (Met) methylation pathway in plants and bacteria (Otte, 2004; Lyon et al., 2011; Williams et al., 2019), a transamination pathway in marine bacteria and algae (Curson et al., 2017, 2018; Zhang et al., 2019), and a decarboxylation pathway in one dinoflagellate (Uchida et al., 2012). The key methylthiohydroxybutyrate *S*-methyltransferase enzyme of the Met transamination pathway is catalyzed by three distinct *S*-adenosyl-Met (SAM)-dependent *S*-methyltransferase enzymes in different organisms. These are DsyB in some alpha-proteobacteria (Curson et al., 2017), DSYB in algae and corals (Curson et al., 2018), and TpMMT in the diatom *Thalassiosira pseudonana* (Kageyama et al., 2018). Recently, the Met methyltransferase, MmtN of the Met methylation pathway was identified in some Gram-positive bacteria, alpha-proteobacteria, and gamma-proteobacteria (Liao and Seebeck, 2019; Williams et al., 2019). Currently, no enzymes of the Met decarboxylation pathway are known. The *dsyB*, *DSYB*, and *mmtN* genes are robust reporters for the ability of an organism to produce DMSP, but the function of TpMMT encoded by the gene *TmMT2* was only confirmed in *T. pseudonana*. Recent analysis of metagenomic data predicts that 1 and 0.1% of surface saltmarsh sediment bacteria contain *dsyB* gene and *mmtN*, respectively (Williams et al., 2019). Furthermore, Williams et al. predicted that ∼10^4^ and 10^8^ bacteria per mL/g of surface seawater or sediment have the genetic potential to produce DMSP. They propose bacteria as significant DMSP producers in both these environments but that their role is much more important in the sediment than the seawater. Curson et al. (2018) found that there were ∼two-fold more algal *DSYB* transcripts than those for the bacterial *dsyB* gene in North Pacific Ocean coastal seawater samples. This supports algae as the major DMSP producers in photic seawater.

DMSP is degraded via the cleavage and demethylation pathways in heterotrophic bacteria and some algae for the cleavage pathway (Figure 1; Johnston, 2015; Zhang et al., 2019). In the DMSP cleavage pathway, eight distinct DMSP lyase enzymes generate DMS and either 3-hydroxypropionate (3-HP) in the case of the DddD enzyme (Todd et al., 2007), or acrylate with DddL (Curson et al., 2008), DddP (Todd et al., 2009), DddQ (Todd et al., 2011), DddY (Curson et al., 2011a, b), DddW (Todd et al., 2012), DddK (Sun et al., 2016; Schnicker et al., 2017), and the algal DMSP lyase Alma1 (Alcolombri et al., 2015). The *ddd* genes are found in a wide range of proteobacteria [mainly alpha-proteobacteria with *Roseobacters* and SAR11 (*Candidatus* Pelagibacter) being important representatives] and some fungi in the case of *dddP* (Todd et al., 2009). The DMSP demethylation pathway that can result in the generation of MeSH (Reisch et al., 2011a) is initiated by the DmdA enzyme that was identified in *Ruegeria pomeroyi* (Howard et al., 2006) and is common in many marine alpha-proteobacteria, including SAR11 bacteria (Reisch et al., 2008, 2011b). Many diverse bacteria that do not always have the capacity to demethylate DMSP (lacking *dmdA* in their genomes) contain *dmdBCD*/*acuH*, which encode enzymes that degrade the product of DMSP demethylation, methylmercaptopropionate (MMPA), to generate MeSH (Shao et al., 2019). Thus, the presence of *dmdBCD* is considered an indicator of MMPA degradation rather than of DMSP. Most previous metagenomic studies of DMSP cycling focus on seawater, particularly surface seawater, to assess the distribution and diversity of DMSP degradation genes (Howard et al., 2006; Moran et al., 2012; Cui et al., 2015; Nowinski et al., 2019). These studies generally report that *dmdA* dominates the seawater DMSP catabolic gene pool, with ∼33% of marine bacteria predicted to contain *dmdA* from SAR11 clade and *Roseobacter* group bacteria (Howard et al., 2006, 2008; Nowinski et al., 2019). To date, no one single DMSP lyase gene is as abundant as *dmdA* in any marine metagenome but in total ∼20% of bacteria are predicted have one of the 7 known bacterial DMSP lyase genes (Curson et al., 2018). DddP is the most abundant environmental DMSP lyase and is often found in the marine *Roseobacter* clade (Cui et al., 2015; Kudo et al., 2018).

Methanethiol can be modified through two different pathways catalyzed by the MeSH oxidase MTO or the MeSH *S*-methylase MddA enzymes (Figure 1). The MeSH degrading enzyme MTO, found in *Thiobacillus*, *Rhodococcus*, and *Hyphomicrobium* strains, oxidizes MeSH to yield formaldehyde (Suylen et al., 1987; Gould and Kanagawa, 1992; Kim et al., 2000; Lee et al., 2002; Eyice et al., 2018). Metagenomics analysis suggested that the *mtoX* gene was widely distributed in seawater (0.4–45.6% of bacteria), freshwater (5.3% of bacteria), soil environments (∼6.3% of bacteria; Eyice et al., 2018), and saltmarsh sediment (4.0%; Carrión et al., 2019). In contrast, MddA was identified in *Pseudomonas deceptionensis* (Carrión et al., 2015, 2017), but many diverse aerobic and anaerobic bacteria and cyanobacteria also contain MddA and likely methylate MeSH to generate DMS (Drotar et al., 1987; Kiene and Hines, 1995; Stets et al., 2004; Carrión et al., 2015). The *mddA* gene is present in varied environmental metagenomes, but thus far has been found to be much more abundant in terrestrial than in marine environments (Carrión et al., 2015, 2017, 2019). For example, 5–76% of bacteria in soil metagenomes were predicted to contain *mddA*, compared to 9.6% of bacteria from surface saltmarsh sediment samples and ≤ 0.5% of bacteria in seawater metagenomes. Another DMSP-independent pathway for the production of DMS is the reduction of DMSO by the DMSO reductase (DMSOR) enzyme present in some marine heterotrophic bacteria and bacteria associated to anaerobic environments (Griebler, 1997; Kappler and Schäfer, 2014).

Some bacteria consume DMS and three DMS-degrading enzymes have been discovered (Figure 1). DMS can be oxidized to generate DMSO by DMS dehydrogenase (DdhA) in, e.g., *Rhodovulum sulfidophilum* (McDevitt et al., 2002), or by trimethylamine monooxygenase (Tmm) in many *Roseobacters* and notably SAR11 bacteria (Lidbury et al., 2016). Some alpha- and beta-proteobacteria and *Actinobacteria* (Visscher and Taylor, 1993; Borodina et al., 2000; Boden et al., 2011) can oxidize DMS to generate MeSH via the DMS monooxygenase enzyme DmoA (Boden et al., 2011). DmoA was predicted to occur in 0.5% of surface saltmarsh bacteria, whereas the DdhA was far more abundant in these samples (13.3% of bacteria), as was Tmm (2.1% of bacteria) (Carrión et al., 2019).

It is clear from Carrión et al. (2019); Wilkening et al. (2019), and Williams et al. (2019) that coastal marine sediments may be important sites for organosulfur cycling. However, very few studies have examined the genetic potential of marine sediments for the cycling of DMSP and related molecules. In this study, we examine marine surface sediment samples from the eastern Chinese marginal sea, i.e., the Bohai Sea and Yellow Sea sediments (BYSS) and the Okinawa Trough hydrothermal field sediment and polymetallic sulfide (OTSP) for their standing stock DMSP concentration, microbial diversity, and genetic potential to cycle organosulfur compounds. Furthermore, we contrast these data to those generated from the surface seawater from the same sites. Very little is known about organosulfur cycling in deep-sea hydrothermal environments. The closest reported study examines the surface water metagenome of Kueishantao shallow-sea hydrothermal field and found that the DMSP demethylase affiliated to *Roseobacter* and SAR11 clade bacteria was present (Tang et al., 2013). Our study shows that: DMSP concentrations were far higher in marine sediment than those in surface waters per unit volume; DMSP-producing bacteria with DsyB were present in most seawater and sediment samples; bacteria with the genetic potential to cleave DMSP generating DMS were far more abundant than those with *dmdA* in all tested sediment, but that *dmdA* was more prominent in surface waters; bacteria with *mddA* were very abundant in marine sediment but not in seawater; DMSP catabolic potential via known pathways was rare in OTSP samples, yet genes for DMS, DMSO, and MeSH cycling pathways were more abundant.

## Materials and Methods

### Study Area and Sampling

**FIGURE 2:**
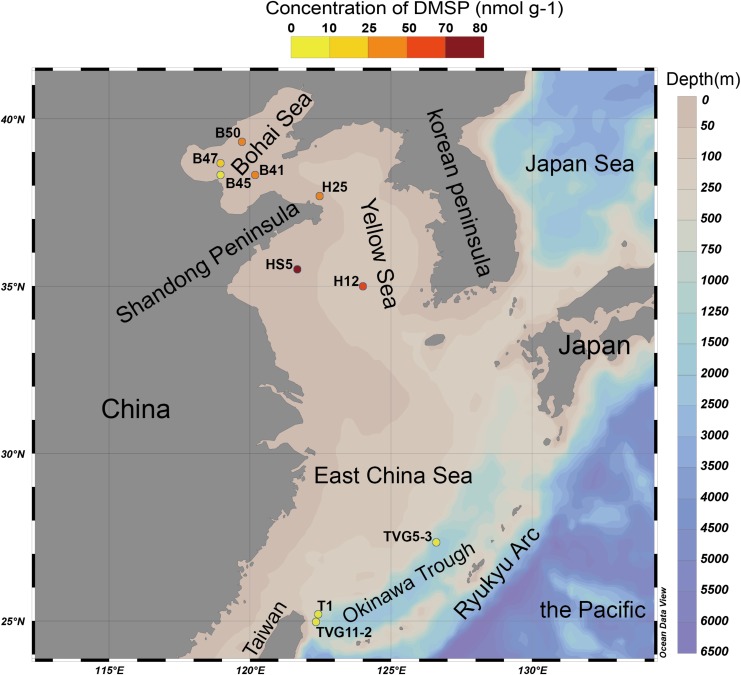
The Eastern Chinese marginal seas sampling stations. “B” refers to Bohai Sea, “H” to Yellow Sea, and “T” to Okinawa Trough. The sites B41, B45, B47, B50, and T1 were sampled only for sediment; sites HS5 and H25 were sampled for water samples (surface and bottom) and sediment samples; and sites TVG11-2 and TVG5-3 were sampled for sediment and polymetallic sulfide. Stations plotted in Ocean Data View (Schlitzer, 2002). The total DMSP concentration in the surface sediment of each site is indicated by the colored circle according to the DMSP concentration scale.

The eastern Chinese marginal seas are a semi-enclosed marginal region of the western Pacific Ocean, and includes the Bohai Sea (BS), the Yellow Sea (YS), and the East China Sea (Figure 2). The mean depths of the BS and the YS are < 20 and ∼50 m, respectively. Both are fed by 30–40 rivers, notably the Yellow River, which empties into the BS. Both seas are surrounded by areas of high population and economic development in China and the Korean Peninsula (Figure 2), with significant effects on their ecosystems (Liu et al., 2011; Xu, 2011).

The Okinawa Trough is located between the East China Sea and the Ryukyu Arc (Figure 2). To date, more than 20 hydrothermal vents have been discovered in this region (Miyazaki et al., 2017b), many of these are in the Iheya ridge hydrothermal field (Stations of T1 and TVG11-2; Figure 2) (Kawagucci et al., 2011). This deep-sea hydrothermal vent environment has mean depths of around 1300 m, and is characterized by a thick blanket of muddy sediment of volcanic sands containing minor sulfide components (Sakai et al., 1990; Gamo et al., 1991; Ishibashi et al., 1995). Other deep-sea (mean depth of 1206 m) hydrothermal vents were found in the Tangyin hydrothermal field (the station of TVG5-3), which is located at the southwest of the Okinawa Trough, close to Taiwan island (Figure 2; Zeng, 2015). This site is characterized by lead-enriched polymetallic sulfides and abundant sulfate minerals (Ishibashi et al., 2015).

The sediment samples (top 5 cm surface sediment, predicted to span zones with the highest DMSP concentration; Wilkening et al., 2019) used in this study were collected in the YS (at sites termed H12, HS5, H25) and the BS (B41, B45, B47, B50) by grab sampler (Cuong and Obbard, 2006) and in the Okinawa Trough hydrothermal field at sites TVG11-2, TVG5-3, and T1 from sediment and polymetallic sulfide (see Figure 2 and Table 1 for locations and details of the sampling stations). The reported maximal photic zone depth of YS is ∼30 m (Jin et al., 2013), and the BS is ∼14 m (Shang et al., 2011). Thus, all sediment samples in this study were taken from below the photic zone, the sampling depths are shown in Table 1. The surface water samples (0 m) and bottom water samples (Table 1) were collected in the YS (H12, HS5) using a Sea logger CTD (Conductivity–Temperature–Depth, Sea-Bird SBE911) rosette water sampler; 20 L Seawater was filtered serially through 3 μm (TSTP, 142 mm, Millipore) and 0.2 μm (GTTP, 142 mm, Millipore) polycarbonate membranes (Zhang et al., 2008). The communities collected on the 3 and 0.2 μm filters were designated as particle-associated and free-living fractions, respectively. All filters were stored in liquid nitrogen onboard and at −80°C in the laboratory. Here, we integrated data from both the > 3μm and 0.2–3 μm seawater fractionations because the difference between these fractions was not the focus of this study.

**TABLE 1:** Data on sediment sampling sites in three different seas.

Station	Sea	Longitude (E)	Latitude (N)	Depth of seabed at sampling site (m)	Depth of sampling (below the sediment surface)	Temperature of seawater (°C)	Date of sampling	DMSP (nmol g^–1^ or nmol L^–1^)	Project
H12	Yellow Sea (sediment)	124.00°	35.00°	72.0	5 cm	23.15	June 30, 2016	62.5 ± 4.8	This work
H12	Yellow Sea (seawater)	124.00°	35.00°	72.0	0 m (surface water) 60 m (bottom water)	23.15	June 30, 2016	ND	This work
HS5	Yellow Sea (sediment)	121.67°	35.50°	39.0	5 cm	23.59	June 30, 2016	72.2 ± 8.6	This work
HS5	Yellow Sea (seawater)	121.67°	35.50°	39.0	0 m (surface water) 30 m (bottom water)	23.59	June 30, 2016	ND	This work
H25	Yellow Sea (sediment)	122.47°	37.69°	51.0	5 cm	18.80	September 11, 2017	25.2 ± 9.3	This work
F8	Yellow Sea (seawater)	124.00°	35.01°	83.0	371 cm	21.84	June 21–July 11, 2013	4.6 (DMSPd) 18.0 (DMSPp)	Yang et al., 2014
G1	Yellow sea (seawater)	121.00°	35.99°	32.0	271 cm	20.77	June 21–July 11, 2013	10.7 (DMSPd) 32.5 (DMSPp)	Yang et al., 2014
F3	Yellow sea (seawater)	121.50°	35.00°	41.0	291 cm	22.65	June 21–July 11, 2013	10.0 (DMSPd) 25.0 (DMSPp)	Yang et al., 2014
B41	Bohai Sea (sediment)	120.18°	38.33°	15.0	5 cm	20.00	September 13, 2017	26.9 ± 4.8	This work
B45	Bohai Sea (sediment)	119.00°	38.32°	24.0	5 cm	24.32	September 13, 2017	9.5 ± 1.4	This work
B47	Bohai Sea (sediment)	118.97°	38.67°	22.0	5cm	23.28	September 14, 2017	13.6 ± 1.0	This work
B50	Bohai Sea (sediment)	119.71°	39.31°	22.0	5 cm	20.09	September 14, 2017	35.4 ± 2.5	This work
TVG5-3	Iheya ridge hydrothermal field of Okinawa Trough	126.59°	27.33°	1549.0	5 cm	ND	June 16, 2016	6.2 ± 0.3 (sediment) 6.5 ± 0.6 (polymetallic sulfide)	This work
TVG11-2	Tangyin hydrothermal field of Okinawa Trough	122.34°	25.03°	1170.0	5 cm	ND	June 17, 2016	6.4 ± 2.6 (sediment) 6.6 ± 2.8 (polymetallic sulfide)	This work
T1	Tangyin hydrothermal field of Okinawa Trough	122.35°	25.04°	1206.0	5 cm	ND	May 27, 2014	6.8 ± 2.3 (sediment)	This work

*ND: not detected.*

### Measurements of DMSP Concentrations in Samples

The total DMSP concentrations in sediment and polymetallic sulfide samples were assessed by measurement of headspace DMS derived from DMSP by alkaline lysis, in 2.0 mL capacity vials (Agilent) containing 0.2 mL liquid samples, by using a flame photometric detector (Agilent 7890A GC fitted with a 7693 autosampler) and a HP-INNOWax 30 m × 0.320 mm capillary column (Agilent Technologies J&W Scientific). Briefly, the sediment or polymetallic sulfide samples (0.1–0.15 g) were mixed with 100 μL ultrapure water; 100 μL of 10 mol L^–1^ NaOH solution was added to the sample-water mixture and was incubated overnight with oscillation at room temperature to allow complete lysis DMSP to DMS. An eight-point calibration curve of DMS standards was used (Liu et al., 2018), and the detection limit for headspace DMS was 0.015 nmol. No steps were taken to account for endogenous DMS in the samples, thus, these assays represent total DMSP plus endogenous DMS. However, according to the previous studies (Nedwell et al., 1994; Kiene and Linn, 2000). DMSP always predominates over DMS in marine samples, thus, it is most likely the majority of DMS detected in this study is derived from DMSP. The DMSP concentrations in surface seawaters were not tested but were reported for the YS previously by Yang et al. (2014).

### Quantification of 16S rRNA Gene in Samples

To compare the number of bacteria in seawater and sediment, quantitative polymerase chain reaction (qPCR) of the 16S rRNA gene was performed on StepOne ABI (Applied Biosystems, Foster City, CA, United States). The abundance of 16S rRNA gene was quantified using the primer set Eub338F (5′-ACTCCTACGGGAGGCAGCAG-3′) and Eub518R (5′-ACTCCTACGGGAGGCAGCAG-3′) (Yin et al., 2013). qPCR reactions were performed in triplicates in a 20 μL system using 10 μL 2 × SYBR Premix Ex Taq II (Takara Bio Inc.), 0.4 μL 50 × ROX reference dye, 0.2–0.4 μM of each primer, 2 μL 1/10 diluted template DNA. The PCR reaction conditions were as described in Liu et al. (2018). Amplification efficiency for the 16S rRNA gene is 0.93, with an *R*^2^ value of 0.99.

### DNA Extraction and Metagenomic Sequencing

Total DNA of seawater and sediment samples were extracted using the method described by Zhou et al. (1996). Briefly, to extract DNA from water (20 L), biomass was washed from half of each filter using 15 mL extraction buffer [100 mM Tris-HCl (pH 8.0), 100 mM sodium EDTA (pH 8.0), 100 mM sodium phosphate (pH 8.0), 1.5 M NaCl, 1% CTAB] before centrifuging at 5000 × *g* for 20 min at room temperature. The concentrated biomass was ground in liquid nitrogen, proteinase K and SDS were added in sequence, and the samples were incubated at 37 and 65°C, respectively, followed by phenol-chloroform extraction. DNA was precipitated with 0.6 volume of isopropanol, washed with 70% ethanol, air dried, and dissolved in TE buffer. The concentration and integrity of genomic DNA were analyzed by a Nanodrop 2000 spectrophotometer (Thermo Scientific, Denmark) and agarose gel electrophoresis (AGE), respectively.

To extract DNA from sediment and polymetallic sulfide, the samples (60 g) were ground in liquid nitrogen for temporary freeze and then collected into a filter containing 15 mL extraction buffer. Proteinase K and SDS were added in sequence with incubation at 37 and 65°C, respectively, followed by centrifugation at 6000 × *g* for 20 min at room temperature. The rest of the protocol was the same as for DNA extraction from water samples.

A total of 16 DNA samples (4 from seawater, 10 from sediments, and 2 from polymetallic sulfide) were sent to BGI (BGI, Shenzhen, China) for metagenomic sequencing. Metagenomic shotgun sequencing was performed on the Illumina HiSeq X-Ten platform, with 2 × 150 bp paired-end reads. First, DNA purity and integrity (no obvious disorganized bands and the main band ≥ 20 kb) were analyzed using AGE and quantification of DNA concentration (≥ 15 ng/μL) and quantity (≥ 1.5 μg) was performed using Qubit. The qualified DNA samples are randomly broken into fragments of approximately 350 bp in length using a Covaris focused ultrasound system. DNA fragments were end-repaired by addition of End Repair Mix and purified by QIA quick PCR Purification Kit (Qiagen). A-Tailing Mix was used to add an adenine at the 3′ end, and then the sequencing linker was ligated to both ends of the DNA fragment. Fragment selection was performed using 2% agarose gel and QIA Quick Gel Extraction kit (QIAGEN), and PCR amplification of several cycle was performed. The PCR product was purified again on a 2% agarose gel and the target fragment was recovered using QIA Quick Gel Extract on kit (QIAGEN). Finally, Agilent 2100 Bioanalyzer and ABI StepOnePlus Real-Time PCR System were used for quality control and quantification of sample libraries. Qualified libraries were sequenced using the Illumina Miseq PE300 platform (MiSeq Reagent Kit v3) at Major bio BiFo-Pharm Technology Co., Ltd., Shanghai, China. Sequences were processed with the pipeline of UPARSE (Edgar, 2013). The clean data basic information of all samples has been shown in Supplementary Table S1.

### Metagenomic Sequence Assembly and Binning

All the raw reads containing > 10% of undefined bases, > 40% of low-quality bases, and that had > 15 bases matching the adapters were removed. The quality-filtered reads were assembled using Megahit (Li et al., 2015) with a kmer size of 49. Contigs > 500 bp were retained. Gene prediction was performed using MetaGeneMark (Zhu et al., 2010) with default parameters^1^. Sequences were clustered at 95% identity and 90% coverage using CD-Hit (Li and Godzik, 2006). The longest sequence of each cluster was selected as a representative sequence of the cluster in order to create a non-redundant robust gene database. Then metagenomics reads of each sample were mapped to the non-redundant gene database using SoapAligner (Qin et al., 2012; Graham et al., 2017). Those sequences with less than two reads mapped were discarded and the resultant quality-filtered sequences were used for downstream analysis. The abundance of each gene was calculated using the calculation method (Supplementary Figure S1) and normalized by the number of mapped reads of the target gene to the gene length.

The initial paired-end reads from these samples were co-assembled using Megahit (Li et al., 2015), the principle of co-assembly is that all 3 and 0.2 μm samples from surface or bottom seawater at the same location in the YS were combined, as were all YS sediment samples, all BS sediment samples, and all Okinawa Trough samples. Subsequently, these reads were mapped to contigs using BWA (Short sequence alignment software; Li and Durbin, 2009), and then, grouped into metagenome assembled genomes (MAGs) by using the very sensitive mode of MetaBAT (Kang et al., 2015). Completeness and contamination of MAGs were assessed using CheckM, and MAGs with a completeness ≥ 80% and contamination ≤ 10% (high completion MAGs) were considered for further analysis. NCBI-nr (released August 2016) protein database and DIAMOND software (Buchfink et al., 2014) were used to determine the taxonomy of MAGs, followed by the MEGAN software (Huson et al., 2007; Liu et al., 2019) (version 4.6) system classification of the lowest common ancestor (LCA) algorithm to ensure the taxonomy. The data on MAGs are shown in Supplementary Table S2.

### Taxonomic Assignment

The filtered genes were compared against the NCBI-nr (released August 2016) protein database using DIAMOND software (Buchfink et al., 2014) BLASTp method, and matches with the lowest e-value minimum alignment results were selected for subsequent analysis. Since each sequence may have multiple alignment results through the above method, the MEGAN software (Huson et al., 2007; Liu et al., 2019) (version 4.6) system classification of the LCA algorithm was used to ensure its biological significance. According to the LCA annotation results and the gene abundance data, the abundance information of each sample at each classification level was obtained (Supplementary Figure S2). The abundance of a species in a sample was calculated by summing the abundances of genes annotated to the species in the sample. The calculation of the relative abundance of taxa possessing a certain DMSP cycling gene was calculated based on the assumption that the abundance of a species equals the total abundances of genes annotated to this species in the same sample.

### Metagenomic Analysis

To explore the genetic potential of DMSP cycling, hidden Markov Model (HMM)-based searches for homologs in metagenome datasets and MAGs were performed using HMMER tools (version 3.1^2^; Kumaresan et al., 2018). Ratified DddD, DddK, DddL, DddP, DddQ, DddY, DddW, Alma1 (*Symbiodinium* and *Emiliania*), DsyB, DSYB, MmtN, DmdA, DmdB, DmdC, DmdD, AcuH, MddA, DmoA, DdhA, Tmm, MTO, and DMSOR sequences were used as training sequences to create the HMM profiles. All the training protein sequences are shown in Supplementary Table S3. Profile HMM-based searches eliminate the bias associated with single sequence BLAST queries (Eddy, 2011). Separate cut-off E-values were confirmed by blasting between functionally verified protein sequences (training sequences; Supplementary Table S4). All retrieved homolog sequences were aligned to the training sequences using the HMM alignment and this was used to construct an approximately maximum likelihood phylogenetic tree inferred using MEGAN v7.0.26 to further ensure the accuracy of the Hmm-based method. Candidate genes were removed if they did not align most closely to the ratified enzymes compared to proteins with different functions. Furthermore, the two databases KEGG and COG were also used to further ensure the reliability of the HMM method for gene function prediction. To estimate the proportion of bacteria and eukaryotes represented in each metagenome, selections of RecA proteins with a cut-off of E ≤ e^–50^ (Curson et al., 2017) and β-Actin proteins with a cut-off of E ≤ e^–60^ were used as probes to BLAST the same metagenome databases. The numbers of genes of interest detected in the metagenome data from each sample can be found in Supplementary Table S5. Additionally, the retrieved DsyB, DSYB, DmdA, and Ddd homolog sequences were aligned to the training sequences using the counterparts HMM alignment and the sequences that could not be sufficiently aligned were discarded, this was used to construct an approximately maximum likelihood phylogenetic tree inferred using FastTree v2.1 (Price et al., 2010). The taxa possessing each candidate gene was found using the taxonomic annotation and the abundance of taxa in each sample was calculated.

### Data Availability

The metagenome data used in this study have been deposited at NCBI/BioProject, the Bio-Project accession numbers of these metagenome data are as follows: YS (PRJNA428417), BS (PRJNA514927), and Okinawa Trough hydrothermal field (PRJNA514953). The accession numbers of MAGs see the Supplementary Table S2. All the training sequences used to create the HMM profiles are available within the Supplementary Materials in the Supplementary Table S3.

## Results

### DMSP Concentrations Are Higher in Marine Sediment From the Bohai Sea, Yellow Sea, and Okinawa Trough Hydrothermal Field Than in Surface Seawater Samples

Marine surface sediment samples from the YS (25.2–72.2 nmol g^–1^) and BS (9.5–35.4 nmol g^–1^) contained relatively high DMSP standing stock concentrations (Table 1). Unfortunately, we did not measure the DMSP levels in the surface waters for these sites on this cruise. However, these sediment values (Table 1) are about two orders of magnitude higher per mass unit than those reported for the surface waters (DMSPd, 4.6–10.7 nmol L^–1^; DMSPp, 18.0–32.5 nmol L^–1^; Table 1) in the YS on an earlier cruise (Yang et al., 2014). The sediment levels are ∼three-fold lower than the DMSP levels reported for surface saltmarsh sediments in Williams et al. (2019). In comparison, the DMSP concentration in OTSP samples (6.2–6.8 nmol g^–1^; Table 1) was ∼10-fold lower than the BYSS samples (Table 1 and Figure 2) and was similar to the levels previously reported for 4.5 km deep surface sediment from the Challenger Deep of the Mariana Trench (Williams et al., 2019). There was no noticeable difference in the DMSP levels of the hydrothermal sediment and polymetallic sulfide samples. Even the OTSP samples had far higher DMSP standing stock concentrations than surface seawater samples (Table 1), suggesting that marine sediments are highly active sites for DMSP cycling. As DMSP is rapidly degraded by abundant DMSP catabolic enzymes in the seawater (Kiene, 1996; Ledyard and Dacey, 1996), it is likely that much of the DMSP in the sediment may be derived from biosynthesis rather than sinking particles. However, further studies measuring the rates of DMSP synthesis and catabolism are required to test this hypothesis.

### The Genetic Potential for DMSP Biosynthesis in Samples

**FIGURE 3:**
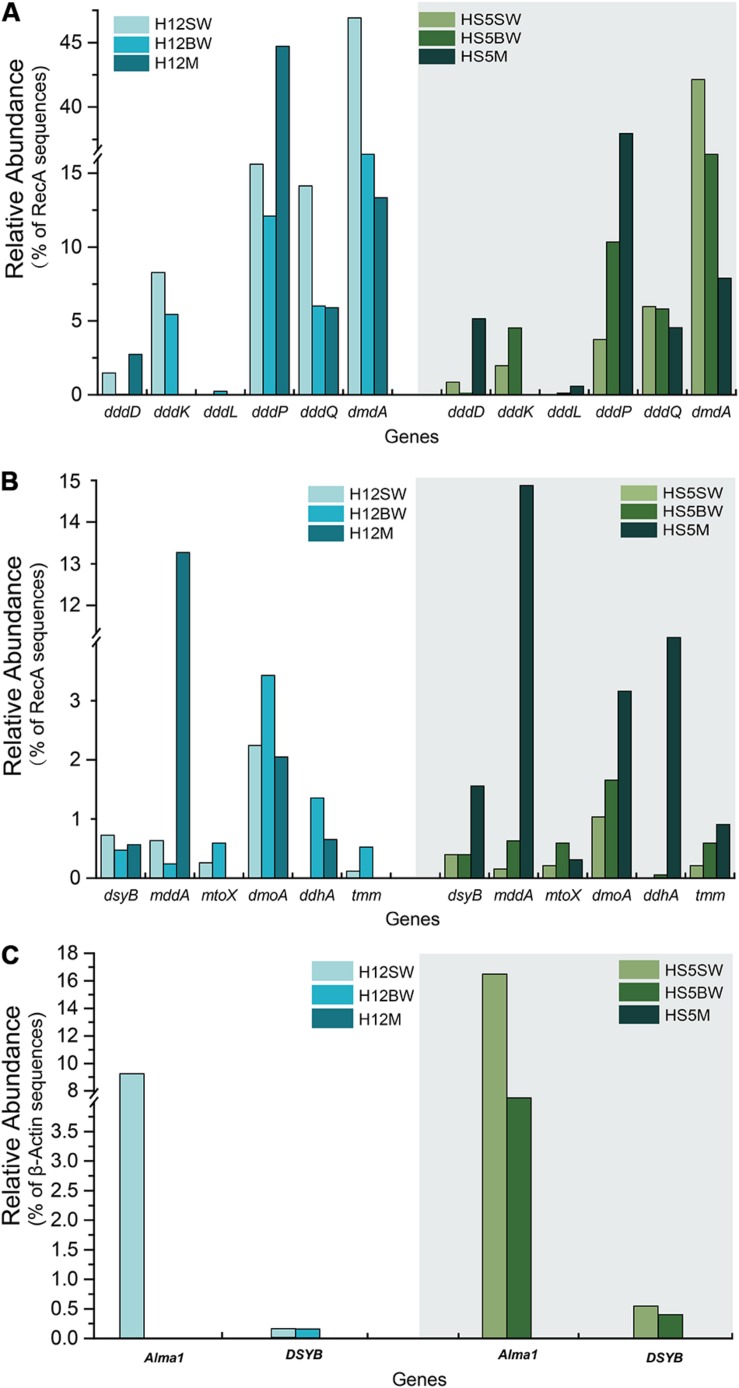
Comparison of relative gene frequencies in sediment and seawater samples from the Yellow Sea. **(A)** The relative abundance of bacterial DMSP catabolic genes (*ddd* and *dmdA* genes) in sediment and water samples from the Yellow Sea. **(B)** The relative abundance of bacterial organosulfur cycling genes in sediment and water in the Yellow Sea. **(C)** The relative abundance of algal genes in sediment and water in the Yellow Sea. SW, surface water; BW, bottom water; M, sediment.

**FIGURE 4:**
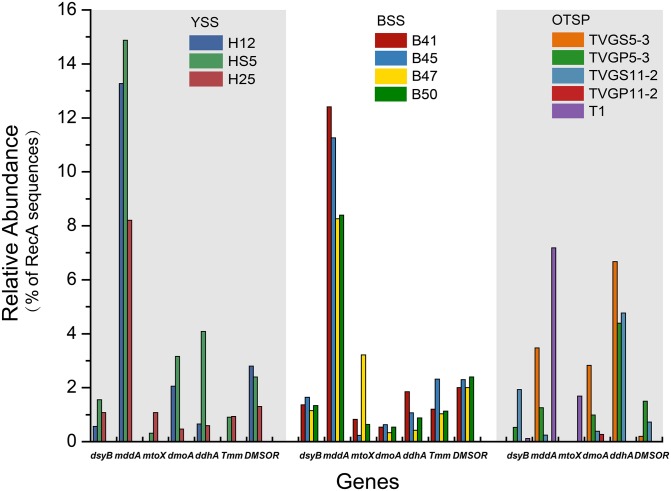
Relative abundances of known genes for the production and cycling of DMSP and related compounds in YSS, BSS, and OTSP sampling stations. YSS, sediments of the Yellow Sea; BSS, sediments of the Bohai Sea; OTSP, sediments and polymetallic sulfides of Okinawa Trough hydrothermal vents; TVGS, sediment samples in Okinawa Trough hydrothermal vents; TVGP, polymetallic sulfide samples in Okinawa Trough hydrothermal vents.

**FIGURE 5:**
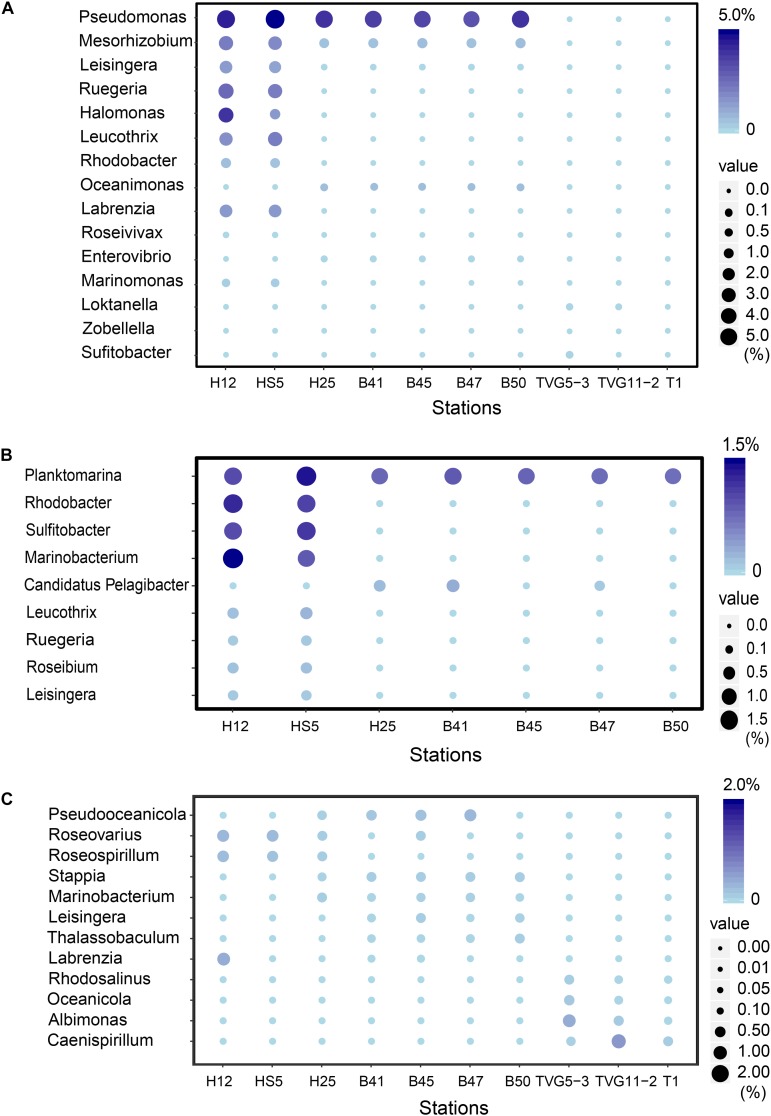
The most abundant bacterial genera predicted to be involved in the production and catabolism of DMSP in all sediment and polymetallic sulfide samples. **(A)** The most abundant bacterial genera possessing “*ddd*” genes. **(B)** The most abundant bacterial genera possessing *dmdA*. **(C)** The most abundant bacterial genera possessing *dsyB*.

Metagenomics was carried out on the surface (SW) and bottom seawater (BW) from sites H12 and HS5 in the YS, and all surface sediment samples taken in this study (Table 1). The metagenomes were examined for the genetic potential to cycle DMSP and related compounds. In both the SW and BW samples *dsyB*, and thus the potential to produce DMSP, was predicted to be present in <1.0% of bacteria (Figure 3B). The dominant seawater *dsyB* genes in metagenome assembled genomes (MAGs) were from alpha-proteobacterial *Roseospirillum* and *Thalassobaculum* bacteria (Supplementary Figures S2,S3). Similar percentages of bacteria with *dsyB* were found in the sediment and polymetallic sulfide samples (average 1.3 and 0.9% in BYSS and OTSP, respectively; Figure 4). 16S rRNA qPCR analysis suggested that there were lower bacterial numbers in SW (1.53 × 10^8^–1.91 × 10^8^ copies L^–1^) than in the BW (6.39 × 10^8^–7.92 × 10^8^ copies L^–1^), but there were far greater bacterial numbers per equivalent mass in the sediment samples (4.62 × 10^7^–1.39 × 10^8^ copies g^–1^). Thus, there are likely far more DMSP-producing bacteria per unit area, playing a more significant role in DMSP synthesis, in the sediment than the seawater environments. Different bacterial *dsyB* genes, clustering with those from *Pseudooceanicola, Roseovarius*, and *Roseospirillum*, dominated in the BYSS sediment compared to those in the seawater (Supplementary Figure S3). The potential DMSP-producing bacteria dominating in the hydrothermal sediments were also distinct with their *dsyB* genes predicted to be in *Caenispirillum*, *Albimonas*, and *Oceanicola* bacteria (Figure 5).

The bacterial *mmtN* gene was absent in all of the metagenome sequences, suggesting that DMSP synthesis via MmtN is not a major pathway in the tested environments. The algal *DSYB* gene was present at similar levels (0.2–0.6% of eukaryotes, Figure 3C) in the SW and BW samples but was not found in the BYSS or OTSP metagenomes. The detected *DSYB* genes were most homologous to those in *Fragilariopsis* and *Alexandrium* (Supplementary Figures S3,S4). This is consistent with Diatom and Dinophyceae phytoplankton being important producers in the tested water samples, but less so in the surface sediments in this study.

### The Genetic Potential for DMSP Catabolism

**FIGURE 6:**
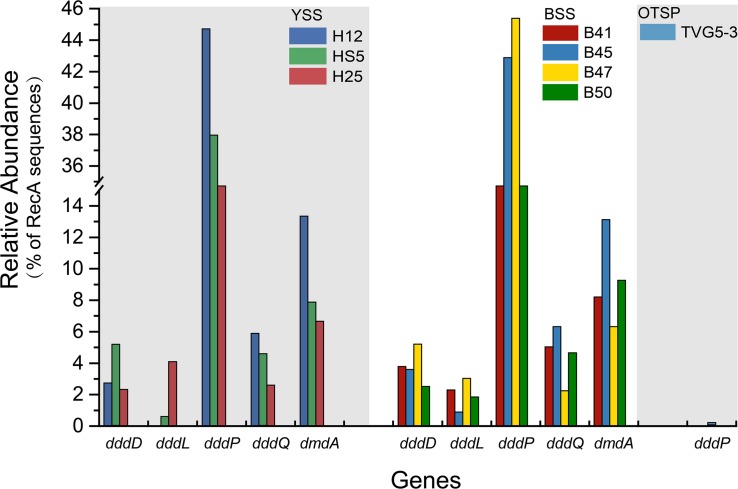
Relative abundances of DMSP catabolic genes in YSS, BSS, and OTSP sampling stations. YSS, sediment of Yellow Sea; BSS, sediment of Bohai Sea; OTSP, sediment and polymetallic sulfide of Okinawa Trough hydrothermal vents.

**FIGURE 7:**
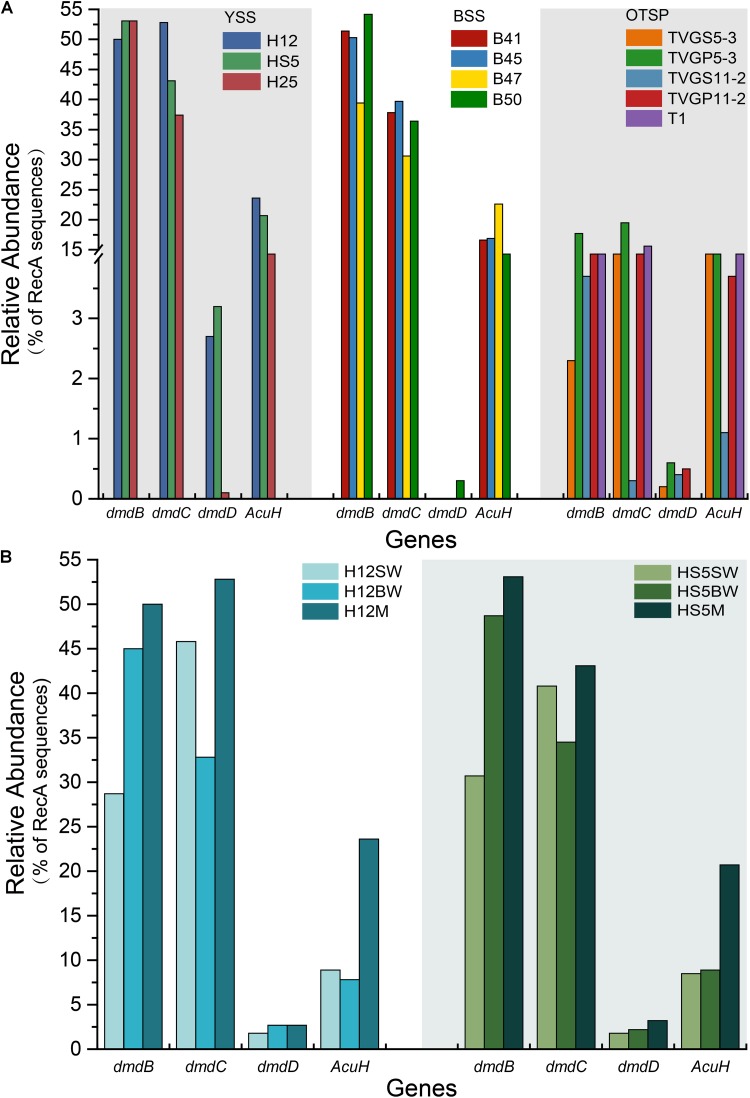
Relative abundances of DMSP demethylation genes in YSS, BSS, and OTSP sampling stations. **(A)** The relative abundance of the bacterial the *dmdBCD* and *acuH* genes in YSS, sediment of Yellow Sea; BSS, sediment of Bohai Sea; OTSP, sediment and polymetallic sulfide of Okinawa Trough hydrothermal vents. **(B)** The relative abundance of the bacterial *dmdBCD* and *acuH* genes in sediment and water in the Yellow Sea.

As expected from previous research (Howard et al., 2006, 2008; Moran et al., 2012; Cui et al., 2015) the *dmdA* gene, conferring the genetic potential to demethylate DMSP, is far more abundant than any other DMSP lyase gene in both the SW (average 44.5%) and BW (average 35.5%) samples (Figure 3A). Interestingly, the percentage of bacteria with *dmdA* declines with seawater depth and is always lowest in the sediment samples (average ∼9.2% in BYSS; Figure 6). Furthermore, *dmdA* is undetectable in the OTSP samples indicating that demethylation is, at best, only a minor component of DMSP catabolism in the Okinawa Trough hydrothermal field (Figure 6). Alternatively, there may be DmdA isozymes in these environments, but there is no evidence that such enzymes exist and further work is needed to test this hypothesis. The genes involved in the catabolism of MMPA and generation of MeSH, *dmdB*, *dmdC*, and *AcuH* had high relative abundances in the investigated samples. In contrast to *dmdA*, the *dmdB* (38.3%), *dmdC* (38.5%), and *AcuH* (8.5%) genes were relatively less abundant in the seawater compared to the YBSS samples, with average abundances of 50.2, 39.7, and 18.1%, respectively (Figure 7A). Furthermore, in the OTSP samples where *dmdA* was undectable, *dmdB* (average 8.9%), *dmdC* (average 11.2%), and *AcuH* (average 7.8%) genes were relatively abundant. These data suggest that MMPA may be a significant source of MeSH in these marine environments and in some cases perhaps is independent of DMSP catabolism, e.g., in the OTSP samples. The *dmdD* (present in up to 2.1% of bacteria) is far less abundant than *acuH* and did not show any significant differences in relative abundance between YS sediment and seawater samples (Figure 7B; *P* > 0.05). These data suggest that AcuH is the major methylthioacryloyl-CoA hydratase responsible for generating MeSH in these environments. The high abundances of *dmdB*, *dmdC*, and *acuH* may be linked to the high plasticity and flexibility of the corresponding enzymes (Reisch et al., 2011a; Bullock et al., 2014; Shao et al., 2019).

In both SW and BW samples, *dddP* was the most abundant DMSP lyase gene, predicted to be present in 4.0–15.6% of bacteria (Figure 3A). In contrast to *dmdA*, the abundance of the *dddP* gene increased in the sediment compared to the seawater samples. In fact, in all sediment samples *dddP* not *dmdA* was the dominant DMSP catabolic gene. Other DMSP lyase genes were also relatively abundant including *dddQ* (6.0–14.1% of bacteria), *dddK* (2.0–8.3% of bacteria), and *dddD* (up to 1.5%) (Figure 3A) in the SW and BW samples. In contrast, *dddL* was only predicted to be in < 0.3% of SW and BW bacteria and no homologs of *dddY* and *dddW* were found in the SW, BW samples, or sediment environments (Figure 3A). The eukaryotic DMSP lyase gene *Alma1*, most similar to *Emiliania huxleyi Alma1*, was detected in the SW and some BW samples, but not in any YS sediment samples, and when detected *Alma1* was predicted to be in ∼4–16% of eukaryotes in these waters (Figure 3C).

There were no obvious differences between the census of DMSP lyase genes in the Yellow Sea sediment (YSS) versus the Bohai Sea sediment (BSS) samples (*P* > 0.05). The *dddP* gene (predicted to be on average in 37.8% of bacteria; Figure 6) was the most abundant bacterial cleavage gene, followed by *dddQ* (average 4.4%), *dddD* (average 3.9%), and *dddL* (average 2.2%) (Figure 6). No *dddY*, *dddK*, and *dddW* homologs were observed in BYSS samples. In contrast to the YSS samples where *Alma1* is absent, very low levels of *Alma1* were detected in 50% of the BSS samples, and this equated to ∼0.8% of eukaryotes in the samples containing this gene (Supplementary Table S5). These data suggest that bacteria and algae are likely important producers of DMS from DMSP in the YS photic waters, but bacteria mainly drive this process in these tested sediments. Alpha-proteobacteria were clearly important in seawater and sediment DMSP catabolic processes. The *dmdA* gene was predominantly in SAR11, SAR116 (*Candidatus* Puniceispirillum), and *Rhodobacterales* bacteria (Supplementary Figure S5) in both seawater and sediment. The *ddd* genes were predominantly in SAR11 and *Rhodobacteraceae* in seawater (Supplementary Figure S3), whereas they were mainly in alpha-proteobacterial *Rhizobiales and Rhodobacterales* bacteria as well as gamma-proteobacterial *Pseudomonadales* bacteria in the sediment (Supplementary Figures S6–S10).

Despite DMSP being detected in hydrothermal field samples and at much higher levels than in the YS seawater, most DMSP catabolic genes, like *dmdA*, were not detected in the majority of hydrothermal OTSP samples. Only one *dddP* sequence was detected in an Iheya ridge sample (Figure 6). This solitary *dddP* gene sequence clustered with the *Sulfitobacter dddP* (Figure 5A). These data suggest that either DMSP lysis, like DMSP demethylation, is not an important process in these hydrothermal sediments or that other unknown DMSP lyases exist in these environments. Further work studying the process rates of DMSP synthesis and degradation is needed to address these questions.

To further predict DMSP metabolic pathways in the investigated samples, we constructed 78 high-quality genomic bins, hereafter referred to as MAGs, from the combined contigs of the samples with a contaminant threshold of 10% and a completeness threshold of 80% (Supplementary Table S2). The *dmdB* and *dmdC* genes are found in most MAGs (Supplementary Table S2), which is likely linked to the high plasticity and flexibility of the enzymes encoded by these two genes. Reisch et al. (2011a) reported that the bacteria possessing *dmdB* and *dmdC* can utilize MMPA to produce MeSH, further supporting MMPA as a likely important source of MeSH in the marine environment. Furthermore, only two of these MAGs (B_bin.4 and B_bin.42; Supplementary Table S2) from sediment samples also contain *dmdA* suggesting that such bacteria contain an isoform DMSP demethylase enzyme or that MMPA may be abundant in these sediments. DMSP lyase genes *dddP* (HS5M_bin.12, B_bin.98, B_bin.4 and B_bin.42), *dddD* (B_bin.33 and B_bin.42), *dddQ* (B_bin.4 and B_bin.42), and *dddL* (B_bin.86) were found in MAGs of the YBSS. These data suggested that DMSP lysis is likely an important process in these sediment samples. However, no DMSP catabolic gene has been found in MAGs of OTSP. These data suggested that DMSP demethylation and cleavage are only a minor component of DMSP cycle in the Okinawa Trough hydrothermal field.

### The Genetic Potential for MeSH Removal in Samples

Methanethiol is biologically modified via two different pathways, i.e., the MeSH-dependent DMS production (MddA) and MeSH oxidation (MTO) pathways. In both the SW and BW, the *mddA* gene was predicted to be present in 0.2∼0.6% of bacteria (Figure 3B). These levels are similar to those predicted for surface ocean bacteria by Carrión et al. (2015). A similar abundance was detected for the *mtoX* gene (0.2∼0.6%) in the same environments. As expected (Carrión et al., 2015, 2019), the *mddA* gene was detected at much higher frequencies in the BYSS samples (average ∼11.0% of bacteria, Figure 4, no significant difference between BS and YS, *P* > 0.05) than in seawater, but *mddA* was always less abundant than the DMSP lyase *dddP* (average 37.8%; Figure 6). In contrast, abundance of the *mtoX* gene was not obviously different in the BYSS (average ∼0.8% of bacteria; Figure 4) compared to the seawater samples. These data are in accordance with Carrión et al. (2019), and suggest that the main biological fate of MeSH in tested BYSS and seawater samples is likely tied to the production of DMS along with the incorporation of MeSH into proteins (Kiene et al., 1999).

In contrast to known DMSP catabolic genes, the *mddA* and *mtoX* genes were detected in all hydrothermal vent sediment samples, with average relative abundances of 2.4 and 1.7% (Figure 4), respectively (no significant difference between sediment and polymetallic sulfide, *P* > 0.05). It would be interesting to know where the MeSH substrate for these processes comes from in these samples where no *dmdA* genes were identified. According to the analysis of MAGs, two MAGs (B_bin.33 and B_bin.40; Supplementary Table S2) from sediment samples contain *mddA* gene, and two contain the MeSH oxidation gene *mtoX* (B_bin.47 and B_bin.12) suggesting that MeSH removal may be an important process in marine sediment.

### The Genetic Potential for DMS Oxidation in Samples

**FIGURE 8:**
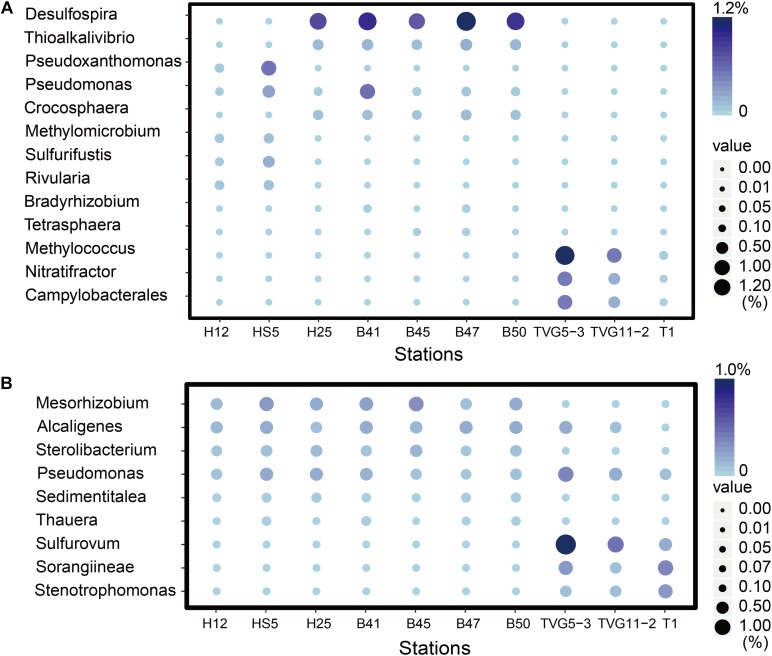
The most abundant genera predicted to oxidize DMS and methylate MeSH in all sediment and polymetallic sulfide samples. **(A)** The most abundant bacterial genera potentially degrading methanethiol (MeSH); MddA, *Desulfospira*, *Thioalkalivibrio*, *Pseudoxanthomonas*, *Pseudomonas*, *Crocosphaera Sulfurifustis*, *Nitratifractor*, *Campylobacterales*; MTO, *Methylomicrobium*, *Rivularia*, *Bradyrhizobium*, *Tetrasphaera*, *Methylococcus*. **(B)** The most abundant genera potentially oxidizing DMS; DmoA, *Alcaligenes*, *Pseudomonas*, *Stenotrophomonas*; DdhA, *Mesorhizobium*, *Sterolibacterium*, *Sorangiineae*, *Pseudomonas*, *Sulfurovum*; Tmm, *Sedimentitalea*, *Pseudomonas*, *Thauera*.

The three key genes for DMS oxidation *dmoA* (1.0∼3.4%), *ddhA* (0.1∼1.4%), and *tmm* (0.1%∼0.6%) were detected in all SW and BW samples (Figure 3B). There was a trend in HS5 samples where the abundance of these three genes increased with water depth and was maximal in the surface sediment, but this was not the case in H12 site samples (Figure 3B). Nevertheless, these DMS oxidation genes were always most abundant in the sediment samples and there were no significant differences between their abundance in the BS and YS sediments (*P* > 0.05). Bacteria with *dmoA*, *ddhA*, and *tmm* were predicted to represent on average ∼1.2, 1.4, and 1.2% of the communities in the BYSS sediment samples (Figure 4). Within seawater samples, the DMS monooxygenase gene *dmoA* was mainly predicted to be in *Belnapia*, *Candidatus* Entotheonella, and *Microbacterium* bacteria (Supplementary Figure S3), but, *Alcaligenes* and *Pseudomonas* within BYSS samples (Figure 8). The profile of bacteria predicted to contain the DMS dehydrogenase gene *ddhA* was also different between the seawater and sediment samples being in *Rhodocyclales* in SW and BW samples (Supplementary Figure S3), whilst in *Mesorhizobium* and *Sterolibacterium* in BYSS samples (Figure 8). Most *tmm* sequences were mapped to *Sedimentitalea* and *Pseudomonas*, and there was no obvious difference between seawater and the BYSS samples (Figure 8). The three genes *mddA* (H12swf_bin.13, H12BWF_bin.49 and OT_bin.42), *ddhA* (B_bin.47 and B_bin.12), and *tmm* (H12bwf_bin.29, H12BWP_bin.26 and B_bin.4) were also found in the MAGs of BS-YS samples. The *dmoA* gene was present at similar abundances in the hydrothermal OTSP samples (average 0.9%) compared to the BYSS samples, but the abundance of *ddhA* slightly increased to 3.1% (average; no significant difference between hydrothermal sediment and polymetallic sulfide, *P* > 0.05). There were no *tmm* genes detected in the OTSP samples. The *dmoA* gene in hydrothermal samples was mainly predicted to be from *Alcaligenes* and *Stenotrophomonas* (Figure 8). The two MAGs with *dmoA* (OT_bin.42 and 66, Supplementary Table S2) are phylogenetically similar to *Stenotrophomonas*, and a homolog of *dmoA* was found in the OT_bin.42 (completeness: 82.91%, contamination: 0). Which is most similar to *Stenotrophomonas* are present in hydrothermal sediments and likely oxidize DMS. *Sorangiineae*, *Pseudomonas*, and *Sulfurovum* were the dominant bacteria predicted to possess *ddhA* in the OTSP. Additionally, homologs of DMSOR (Satoh and Kurihara, 1987; Mcewan et al., 1991; Sambasivarao and Weiner, 1991) were predicted to be in BYSS (1.3∼2.8%) and OTSP bacteria (0.2∼1.5%; Figure 5), but this gene was absent in the seawater metagenome samples. There was no significant difference in the abundance of DMSOR and DMS oxidation genes in the BYSS samples. However, in the hydrothermal samples, DMS oxidation genes were far more abundant (*P* > 0.05). These data suggest that DMS oxidation processes occur throughout the water column and together with DMSO reduction in sediment samples but further experiments involving process measurement and RNA/protein work are required to prove this.

## Discussion

To date, most of the metagenomic studies on DMSP cycling have focused on seawater (Howard et al., 2006, 2008; Todd et al., 2009; Kudo et al., 2018), while only very few study sediments (Visscher et al., 1995) and these are heavily focused on saltmarsh sediments (Carrión et al., 2019; Williams et al., 2019). Such studies are focused on bacteria since the vast majority of known genes involved in the cycling of DMSP and related compounds were discovered in bacteria and are predominantly bacterial, with a few exceptions notably *Alma1*, *dddP* and *DSYB*, and *TpMT2* (Curson et al., 2011a, 2017, 2008; Johnston, 2015; Zhang et al., 2019), which occur in eukaryotes. Here we examined the metabolic potential to cycle DMSP and related compounds in marine and hydrothermal surface sediment versus surface waters to provide insights in the organisms with the genetic potential to drive these processes.

### BYSS Sediment Likely Supports Bacteria Cycling DMSP and Related Compounds

The data in this study show that DMSP is an abundant molecule in BYSS, far more so than in any seawater sample from these YS study sites (Yang et al., 2014). BYSS samples had similar proportions of bacteria with the genetic potential (containing *dsyB*) to synthesize DMSP (1.3%) to those in the tested seawater (*P* > 0.05; Figure 3B). However, considering the far higher numbers of bacteria in sediment (4.62 × 10^7^–1.39 × 10^8^ 16S rRNA gene copies g^–1^) than in seawater (1.53 × 10^8^–7.92 × 10^8^ 16S rRNA gene copies L^–1^) reflected in 16S rRNA qPCR analysis, there is likely a far higher bacterial biomass potentially synthesizing DMSP in the sediment than in any of the water column samples per equivalent sample mass. Thus, the BYSS bacteria are likely making a far larger contribution to the total DMSP levels in this environment than those in the photic seawater samples. *Rhodobacteraceae* bacteria containing *dsyB* were the most likely bacterial producers of DMSP in both the seawater and sediment samples. The *mmtN* gene was not detected in any samples suggesting that DMSP synthesis via MmtN was not a major pathway in the tested environments. Phytoplankton, whose *DSYB* genes were detected in the water column at low levels, are likely the major contributors to DMSP production in this environment. In contrast, the BYSS sediments are likely aphotic and their metagenomes contained no algal DSYB sequences suggesting that these eukaryotes play a less significant role in sediment DMSP production. Further work studying the transcription and/or protein abundance of the different DMSP synthesis genes products is required to test these hypotheses. It is possible that DMSP in the sediment is largely derived from sinking particles and not from synthesis. Considering that DMSP is rapidly degraded in seawater (Kiene, 1996; Ledyard and Dacey, 1996) and the abundance of DMSP catabolic genes in the seawater samples, it is likely that much of the DMSP in the sediment is a consequence of synthesis. Another important point to be mindful of is that when determining DMSP in seawater and sediment by alkaline hydrolysis, we actually measure the DMS derived from DMSP plus the endogenous DMS already in the sample. Unfortunately, we did not measure the endogenous DMS in our environmental samples, thus have no way of knowing the proportion of each these two influential compounds. In seawater samples, ∼9% of DMSP plus DMS was apportioned to volitiles (Kiene and Linn, 2000) and in sediments endogenous DMS can be undetectable (Carrión et al., 2017, 2019) or up to ∼four-fold lower than DMSP (Nedwell et al., 1994). Given DMSP always predominates over DMS in marine samples, we believe that most of the DMS detected in DMSP plus DMS samples are likely to derive from DMSP. However, it is possible that the levels of endogenous DMS were quite different between samples, for instance, in the hydrothermal field samples that are rich in hydrogen sulfide (H_2_S), H_2_S methylation to produce MeSH, and further methylation to DMS by, e.g., MddA, may be more prominent. It is important in future studies that precautions are taken to account for endogenous DMS, together with synthesis and catabolic rate assays to better predict the organosulfur cycling in these environments.

The potential to catabolize DMSP was abundant in all seawater and BYSS samples. DMSP demethylation appeared to be the dominant pathway in the seawater samples with *dmdA* predicted to be present in ∼40.0% of bacteria, which is consistent with the studies of Howard et al. (2006, 2008), Cui et al. (2015), and Steiner et al. (2019). Seawater bacteria with the potential to cleave DMSP were also abundant with *dddP* being the most abundant DMSP lyase gene (4.0–15.6%) but *dddQ* (6.0–14.1%) and *dddK* (8.3%) were also well represented. Interestingly, the relative abundance of *dmdA* decreased with water depth and was fourfold less abundant in BYSS than in seawater samples. Given the likely higher numbers of bacteria in the sediment, the decrease in *dmdA* relative abundance may not result in such a dramatic decrease in bacteria with the potential to demethylate DMSP. However, in the BYSS samples, *dmdA* abundance was much lower than that of *dddP* (*P* < 0.01; Figure 6). Therefore, it is likely that there is a sediment shift where DMSP cleavage may be the major sediment DMSP catabolic pathway as appose to DMSP demethylation in the seawater (Kiene and Linn, 2000; Kiene et al., 2000). Remarkably, the relative abundance of *dddP* in sediment was almost threefold more than in seawater (*P* < 0.01; Figure 3A). The reason for this shift is unknown, but it may be a consequence of the reduction in the abundance of SAR11 in the sediment and an increase in gamma-proteobacteria such as *Pseudomonas* containing *dddP*, or perhaps to the increased DMSP levels experienced in these samples. It was noteworthy that BYSS bacteria predicted to contain *dddP*, *Pseudomonas*, *Mesorhizobium*, and *Leisingera*, are distinct from previous studies that showed the *Roseobacter* clade to be the major bacteria possessing *dddP* (Todd et al., 2009; Varaljay, 2012; Zeng et al., 2016).

Given the abundance of DMSP catabolic genes in seawater and sediment, there are likely to be catabolites, such as DMS and MeSH available in these environments. None of these DMSP metabolites were measured in this study. It is clear that the genetic potential to methylate MeSH through *mddA* is ∼30-fold higher in BYSS than in seawater samples. Thus, the MeSH-dependent DMS production pathway is likely an important process in BYSS samples, but it is not so significant in seawater samples. Perhaps this is a consequence of DMSP and/or H_2_S (both MeSH precursors), being available at lower levels in the seawater compared to the sediment and thus the likely lower levels of MeSH being generated in seawater that is mainly incorporated into proteins. The relative abundance of *mddA* seen in seawater and sediment samples here are similar to those reported in Carrión et al. (2015, 2019). The *mddA* gene is found in a wide range of bacterial taxa which vary dependent on the environment of study. For example, in soil samples, *Mycobacterium*, *Bradyrhizobium*, *Cyanothece*, and *Pseudomonas* bacteria with *mddA* are abundant (Carrión et al., 2015, 2017); whereas *Rhodopseudomonas* and *Thioalkalivibrio* were the main taxa containing *mddA* in saltmarsh sediment (Carrión et al., 2019). In BYSS samples, the main bacteria containing *mddA* were predicted to be *Desulfospira*, *Thioalkalivibrio*, *Crocosphaera*, and *Pseudomonas* (Figure 5). Carrión et al. (2017, 2019) found that seawater and marine and saltmarsh sediments only generated DMS when samples were incubated with MeSH. This indicates that ordinarily much of the DMS generated from these processes is removed by other microbial processes. Indeed, *dmoA*, *tmm*, and *ddhA* genes were also relatively abundant in the marine samples and encode for enzymes that modify and thus remove DMS.

In general, this metagenomics study has shown that there is a very significant potential to catabolize DMSP and its catabolites in both BYSS and seawater samples, but there is a switch-over in terms of the dominant genes and potentially their catabolic pathways between these environments—DMSP demethylation dominating in photic waters to DMSP lysis and MeSH-dependent DMS production in BYSS. Given the vastly higher DMSP concentration and numbers of bacteria in sediment, it is likely that the BYSS environments are centers of high activity for organosulfur cycling. All of which warrant further future investigation.

### The Okinawa Trough Hydrothermal Field Has Its Own Specific DMSP Cycle

This study represents the first to analyze organosulfur cycling potential in deep sea hydrothermal sediment samples by metagenomics. DMSP levels were 10-fold lower in the OTSP hydrothermal sediment than they were in the BYSS samples, but these were still far higher than those in the seawater samples. OTSP DMSP levels were similar to 4.5 km deep ocean sediment samples assayed in Williams et al. (2019), perhaps indicating that depth may be a factor in the reduced DMSP sediment concentration. Further studies of deep ocean sites are required to test this hypothesis. The profile of DMSP synthesis and catabolic potential was very different in the BYSS and OTSP samples. In studies to date on seawater and saltmarsh sediment samples, DMSP synthesis genes were always far less abundant than those for catabolism (Curson et al., 2017, 2018; Williams et al., 2019). In OSTP samples, the reverse was found to be the case, with *dsyB* being more abundant than that of the only detected DMSP catabolic gene (*dddP*; Figure 5). The abundance of *dsyB* did not change between the BYSS and OTSP samples. However, *dddP* was ∼360-fold reduced in its abundance (to 0.1%) compared to BYSS samples. To our knowledge, this is the first report of a marine metagenomics study, where the samples had appreciable DMSP levels, but lack a *dmdA* homolog. Previous studies have shown that DMSP demethylation is the major bacterial DMSP catabolic pathway in seawater environments (Kiene et al., 1999; Niki et al., 2000; Zubkov et al., 2001), accounting for ∼70% DMSP catabolism (Kiene and Linn, 2000). The data presented here suggest either that (i) DMSP catabolism is not an important process in these hydrothermal samples or (ii) novel DMSP lyases and/or DMSP demethylase enzymes exist in hydrothermal sediment samples. We know that there are likely other DMSP lyases to be identified in bacteria, since Liu et al. (2016) identified bacteria from East China Sea seawater with DMSP lyase activity, but which lack known *ddd* and *Alma1* genes (Liu et al., 2018). In contrast, González et al. (2019) considered it would be highly unlikely to find an environmentally significant alternative enzyme to DmdA that would carry out the DMSP demethylation reaction. Further work involving process measurements from hydrothermal samples is required to determine if DMSP cleavage and/or demethylation is an important process in this environment, but these data indicate they are not. It is also possible that DMSP plays a more important role in environmental stress tolerance in the microbial communities of OTSP samples which experience considerably higher temperature and hydrostatic pressure than those of the shallower BYSS samples.

The *mddA* gene is far more abundant than *dddP* in OTSP samples (Figures 4, 5), suggesting that in these samples, MeSH may be more important for the generation of DMS than DMSP in such deep-sea hydrothermal sediment and polymetallic sulfide environments. If this is the case, a key question is where is the MeSH coming from in these hydrothermal sediments considering the DMSP demethylation is absent? MeSH can be produced from the methylation of H_2_S (Drotar et al., 1987) or degradation of Met and S-methylcysteine (Bremner and Steele, 1978; Ferchichi et al., 1985; Taylor and Kiene, 1989). Okinawa Trough hydrothermal vents have been reported to be rich in H_2_S (Miyazaki et al., 2017a), additionally, Kawagucci et al. (2013) found that H_2_S content reached 4.5 mM kg^–1^ in hydrothermal sediments in the northern Iheya. Therefore, we propose that much of the MeSH in the Okinawa hydrothermal field is likely to be produced from H_2_S methylation. Alternatively, given that the *dmdBC* and *acuH* genes are relatively abundant in the OTSP samples, it is possible that MMPA is also an important source of MeSH. Future studies should assay the availability of MMPA in diverse marine environments to determine its potential importance. These hydrothermal sediments present a case similar to those in terrestrial soil environments where MddA-dependent DMS pathways are more abundant than those for DMSP cleavage. A major difference though is that the hydrothermal samples studied here are marine and contain DMSP at appreciable levels. Further studies are required to determine if the MeSH methylation pathway is important in the OTSP environment.

The DMS oxidation genes *ddhA* and *dmoA*, but not *tmm* are also relatively abundant in OTSP samples further supporting the hypothesis that DMS is an important metabolite in these samples. The DMS dehydrogenase gene *ddhA* is more abundant than the DMS monooxygenase gene *dmoA* in these samples and this is in agreement with the findings of Carrión et al. (2019) working on saltmarsh sediment. Together these data support the existence of a unique organosulfur cycling profile in the deep ocean hydrothermal sediments to those in BYSS samples.

### General Limitations of This Study

The sediment sampling regime in this study combined 0–5 cm deep sediment, which likely spanned large gradients in DMSP and DMS (Wilkening et al., 2019; Williams et al., 2019), oxygen, and diverse microbes. For this study, we have to consider these together, thus, the DMSP plus DMS content and microbial community data will be an average not reflecting that in the different sediment zones. For example, the DMSP and DMS concentration is likely to be far higher in the top ∼1 cm than in the deeper anoxic zone (Wilkening et al., 2019; Williams et al., 2019), which will likely have a distinct microbial profile. Nevertheless, the data presented here are convincing that the tested sediments are centers of high microbial organosulfur cycling activity. It would be interesting to study more precisely how microbial organosulfur cycling changes within the different sediment zones.

Another major limitation of this metagenomics study is that it is all only predictive and lacks process and/or data on the transcription and translation of the key organosulfur cycling genes discussed. Additionally, the metagenomic data presented here have no analytical replication and thus only provide a snapshot of metabolic potential. However, it does provide useful insights and hypotheses on organosulfur cycling in marine and hydrothermal sediments that warrant further investigation in the future.

## Data Availability Statement

The datasets generated for this study can be found in the Bio-Project of these metagenome data as follows: Yellow Sea (PRJNA428417), Bohai Sea (PRJNA514927), and Okinawa Trough hydrothermal field (PRJNA514953).

## Author Contributions

X-HZ and JT designed the experiments, analyzed the data, and wrote the manuscript. DS measured the DMSP concentration, did the quantification of 16S rRNA genes, performed the metagenomic analysis, analyzed the data, and wrote the manuscript. JL, YHZ, SZ, and YFZ analyzed the data and wrote the manuscript. HZ performed the metagenomic sequence assembly and binning. MY extracted DNA and performed metagenomic sequencing for hydrothermal field samples. All authors edited and approved the final manuscript.

## Conflict of Interest

The authors declare that the research was conducted in the absence of any commercial or financial relationships that could be construed as a potential conflict of interest.

## Footnotes

1 http://exon.gatech.edu/GeneMark/meta_gmhmmp.cgi

2 http://hmmer.janelia.org/
