# Supplementary material for: Metagenomic Insights Into the Cycling of Dimethylsulfoniopropionate and Related Molecules in the Eastern China Marginal Seas
Source: Front Microbiol. 2020 Feb 18;11:157. doi: 10.3389/fmicb.2020.00157 (PMC7039863; doi:10.3389/fmicb.2020.00157)
Supplement: Supplementary file 1 [file Presentation_1.pdf]

Supplementary Materials

Supplementary Tables

Supplementary Table 1. The Metagenome clean data basic information of all samples

Sample	Clean reads	Contig Number	Assembly Length (bp)	N50 (bp)	N90 (bp)	Average Size (bp)
H12swf	18,102.80	445,562	484,836,788	1,144	559	1,088.15
H12swp	17,146.84	697,891	577,983,922	797	537	828.19
H12bwf	17,284.13	618,026	624,733,236	1,029	558	1,010.85
H12bwp	19,123.78	530,280	486,698,637	895	544	917.81
H12m	31,111.29	629,979	476,040,823	714	527	755.65
HS5swf	24,185.83	863,242	850,874,056	985	555	985.67
HS5swp	19,029.69	889,673	769,763,410	843	541	865.22
HS5bwf	21,933.21	673,037	695,929,207	1,058	557	1,034.01
HS5bwp	20,299.88	593,214	564,627,774	946	548	951.81
HS5m	27,270.10	774,309	641,952,542	786	534	829.07
H25	106,194,046	145,672	145,893,739	994	551	1001
B41	80,741,206	32,622	31,980,432	999	562	980
B45	81,015,472	46,645	47,686,773	1041	564	1022
B47	85,342,684	29,036	23,917,017	793	540	823
B50	116,539,140	212,671	222,127,826	1061	557	1044
T1	86,968,070	135,574	79,410,337	587	348	586
TVG5-3	70,534,322	3,923	2,661,870	638	520	678
TVG11-2	81,951,240	33,259	24,135,629	701	529	725
TVGS5-3	119,612,348	404,396	447,323,409	1,166	565	1106
TVGS11-2	113,860,692	109,320	101,722,206	923	551	930

Supplementary Table 2. Information of high completion MAGs

Bins	completeness(%)	contamination (%)	Taxonomy	Genes involved in DMSP cycling	Accession numbers
H12bwf_bin.29	91.45	2.14	k_Bacteria; p_Actinobacteria; c_Actinobacteria	*dddP, dmdB, dmdC, tmm*	SAMN13828863
H12bwf_bin.44	90.94	4.21	k_Bacteria; p_Bacteroidetes	*acuH, dmdD*	SAMN13828865
H12bwp_bin.25	94.02	1.68	k_Bacteria; p_Planctomycetes; c_Planctomycetacia	*dmdD*	SAMN13828867
H12swf_bin.13	97.35	1.45	k_Bacteria; p_Proteobacteria; c_Alphaproteobacteria	*acuH, dmdB, dmdC, dmoA*	SAMN13828871
H12swf_bin.4	96.96	0.68	k_Bacteria; p_Verrucomicrobia		SAMN13828870
HS5bwf_bin.44	95.54	4.08	k_Bacteria; p_Verrucomicrobia; c_Verrucomicrobiae; o_Verrucomicrobiales; f_Verrucomicrobiaceae		SAMN13828881
HS5bwp_bin.22	95.33	3.91	k_Bacteria; p_Verrucomicrobia; c_Verrucomicrobiae; o_Verrucomicrobiales; f_Verrucomicrobiaceae		SAMN13828882
HS5bwp_bin.32	93.96	4.66	k_Bacteria; p_Planctomycetes; c_Planctomycetacia	*dmdD*	SAMN13828884
HS5swf_bin.18	93.03	1.78	k_Bacteria; p_Proteobacteria; c_Alphaproteobacteria	*acuH, dmdB, dmdC*	SAMN13828889
HS5swf_bin.3	92.26	4.73	k_Bacteria;p_Bacteroidetes	*acuH, dmdD*	SAMN13828886
HS5swf_bin.30	90.12	2.31	k_Bacteria;p_Proteobacteria	*dmdB, dmdC*	SAMN13828891
HS5swf_bin.63	90.21	2.57	k_Bacteria;p_Bacteroidetes	*acuH*	SAMN13828893
HS5swp_bin.4	90.47	0.77	k_Bacteria;p_Planctomycetes;c_Planctomycetacia		SAMN13828895
H12BWF_bin.23	89.69	3.08	k_Bacteria; p_Chloroflexi; c_Dehalococcoidia; o_Dehalococcoidales	*dddP*	SAMN13828862
HS5SWF_bin.65	89.41	0.54	k_Bacteria; p_Bacteroidetes		SAMN13828894
H12SWP_bin.13	89.34	0.11	k_Bacteria; p_Proteobacteria; c_Gammaproteobacteria	*dmdB, dmdC*	SAMN13828874
H12BWP_bin.35	88.89	0.63	k_Bacteria; p__Bacteroidetes		SAMN13828869
HS5BWF_bin.23	88.65	3.23	k_Bacteria;p_Planctomycetes; c_Planctomycetacia	*dmdD*	SAMN13828878
HS5BWP_bin.26	87.63	0.36	k_Bacteria; p_Bacteroidetes		SAMN13828883
HS5SWF_bin.26	87.1	3.06	k_Bacteria; p_Bacteroidetes	*dddP, dddQ, dmdA, dmdB, dmdC*	SAMN13828890
H12BWF_bin.34	86.55	4.8	k_Bacteria; p_Verrucomicrobia; c_Verrucomicrobiae;		SAMN13828864

			o_Verrucomicrobiales		
H12BWP_bin.26	86.52	4.37	k_Bacteria; p_Actinobacteria; c_Actinobacteria	*dddP, dmdB, dmdC, tmm*	SAMN13828868
HS5BWF_bin.2	85.25	2.98	k_Bacteria; p_Proteobacteria; c_Alphaproteobacteria; o_Rhodobacterales	*acuH, dmdB, dmdC*	SAMN13828876
HS5BWF_bin.36	85.04	0.8	k_Archaea; p_Euryarchaeota	*AcuH*	SAMN13828880
H12SWP_bin.12	84.78	1.55	k_Bacteria; Cyanobacteria; o_Synechococcales		SAMN13828873
H12SWP_bin.2	84.02	3	k_Bacteria; p_Bacteroidetes		SAMN13828872
HS5BWF_bin.1	84	2.4	k_Archaea; p_Euryarchaeota		SAMN13828875
H12BWF_bin.49	83.82	8.97	k_Bacteria; p_Actinobacteria; c_Actinobacteria	*dmdB, dmdC, dmoA*	SAMN13828866
H12BWF_bin.3	83.69	1.61	k_Bacteria; p_Planctomycetes; c_Planctomycetacia	*dmdD*	SAMN13828858
HS5SWF_bin.35	83.33	6.8	k_Bacteria; p_Proteobacteria	*dmdB, dmdC*	SAMN13828892
HS5SWF_bin.10	83.16	4.29	k_Bacteria; p_Proteobacteria	*dmdB, dmdC*	SAMN13828887
HS5SWF_bin.12	82.39	4.59	k_Bacteria; p_Proteobacteria; c_Alphaproteobacteria	*AcuH, dmdB, dmdC*	SAMN13828888
H12BWF_bin.21	82.29	2.2	k_Bacteria	*acuH, dmdB*	SAMN13828861
H12BWF_bin.10	82.08	0	k_Bacteria	*dmdC*	SAMN13828859
H12BWF_bin.14	81.2	2.4	k_Archaea; p_Euryarchaeota		SAMN13828860
HS5BWF_bin.10	80.73	0.54	k_Bacteria; p_Actinobacteria; c_Actinobacteria; o_Actinomycetales	*acuH, dmdB*	SAMN13828877
HS5BWF_bin.30	80.56	0.36	k_Bacteria; p_Bacteroidetes		SAMN13828879
HS5M_bin.12	80.14	8.84	k_Bacteria; p_Proteobacteria	*acuH, dddP, dmdB, dmdC*	SAMN13828885
B_bin.47	95	2.9	k_Bacteria; p_Proteobacteria; c_Gammaproteobacteria	*ddhA*, *dmdB, dmdC, mtoX*	SAMN13826822
B_bin.98	94.84	8.11	k_Bacteria; p_Proteobacteria; c_Gammaproteobacteria	*acuH, dddP, dmdB, dmdC, DMSOR*	SAMN13826828
B_bin.4	91.87	2.7	k_Bacteria; p_Proteobacteria; c_Gammaproteobacteria	*acuH, dddP, dddQ, dmdA, dmdB, dmdC, tmm*	SAMN13826814
B_bin.33	90.91	2	k_Bacteria	*acuH, dddD, dmdB, mddA*	SAMN13826819
B_bin.18	90.88	0.72	k_Bacteria		SAMN13826817
B_bin.9	90.27	2.02	k_Bacteria; p_Proteobacteria; c_Gammaproteobacteria	*dmdB, dmdC, DMSOR*	SAMN13826815
B_bin.42	90.04	6.85	k_Bacteria; p_Proteobacteria; c_Alphaproteobacteria	*acuH, dddD, dddP, dddQ,*	SAMN13826821

				dmdA, dmdB, dmdC	
B_bin.62	87.69	2.69	k_Bacteria; p_Proteobacteria; c_Gammaproteobacteria	*dmdB,*	SAMN13826823
B_bin.79	87.54	1.94	k_Archaea		SAMN13826824
B_bin.86	87.05	2.76	k_Bacteria; p_Proteobacteria; c_Gammaproteobacteria	*dddL, dmdB, dmdC, dmdD,*	SAMN13826825
B_bin.40	86.69	2.64	k_Bacteria; p_Proteobacteria; c_Gammaproteobacteria	*dmdB, mddA*	SAMN13826820
B_bin.93	86.6	1.36	k_Bacteria		SAMN13826827
B_bin.87	86.14	3.58	k_Bacteria; p_Proteobacteria; c_Gammaproteobacteria	*dmdC*	SAMN13826826
B_bin.24	86.65	2.55	k_Bacteria; p_Proteobacteria; c_Gammaproteobacteria	*dmdB, dmdC*	SAMN13826818
B_bin.12	81.84	6.47	k_Bacteria; p_Proteobacteria; c_Gammaproteobacteria	*ddhA, mtoX*	SAMN13826816
OT_bin.97	97.44	4.27	Wang et al., unpublished		SAMN13826949
OT_bin.31	96.7	2.75	Wang et al., unpublished		SAMN13826934
OT_bin.83	95.31	0.84	Wang et al., unpublished		SAMN13826947
OT_bin.105	93.64	1.62	Wang et al., unpublished		SAMN13826950
OT_bin.72	95.3	8.78	Wang et al., unpublished	*dmdB*	SAMN13826943
OT_bin.123	92.21	0.75	Wang et al., unpublished	*dmdB*	SAMN13826952
OT_bin.67	92.02	4.25	Wang et al., unpublished	*dmdB*	SAMN13826941
OT_bin.92	91.48	2.27	Wang et al., unpublished		SAMN13826948
OT_bin.74	90.91	0	Wang et al., unpublished		SAMN13826944
OT_bin.119	90.34	0.84	Wang et al., unpublished	*dmdC*	SAMN13826951
OT_bin.57	89.83	2.7	Wang et al., unpublished		SAMN13826938
OT_bin.35	89.78	3.85	Wang et al., unpublished		SAMN13826935
OT_bin.50	88.18	2.91	Wang et al., unpublished		SAMN13826937
OT_bin.18	87.94	2.44	Wang et al., unpublished	*dmdB, dmdC*	SAMN13826933
OT_bin.126	85.61	3.35	Wang et al., unpublished	*dmdB, dmdC*	SAMN13826953
OT_bin.78	84.4	2.2	Wang et al., unpublished		SAMN13826946
OT_bin.66	84.38	1.35	Wang et al., unpublished	*dmdB, dmdC*	SAMN13826940
OT_bin.128	83.94	0	Wang et al., unpublished		SAMN13826955
OT_bin.42	82.91	0	Wang et al., unpublished	*dmoA*	SAMN13826936
OT_bin.127	82.78	4.23	Wang et al., unpublished	*acuH*	SAMN13826954
OT_bin.77	82.62	0.24	Wang et al., unpublished		SAMN13826945
OT_bin.62	82.17	5.08	Wang et al., unpublished	*dmdB*	SAMN13826939
OT_bin.6	81.18	5.08	Wang et al., unpublished	*dmdB, dmdC*	SAMN13826931
OT_bin.15	80.44	0	Wang et al., unpublished		SAMN13826932
OT_bin.69	80.25	5.04	Wang et al., unpublished		SAMN13826942

H: Yellow Sea; B: Bohai Sea; OT: Okinawa Trough; swf: surface water free-living; swp: surface water particle; bwf: bottom water free-living; bwp: bottom water particle; m: sediment; k: kingdom; p: phylum; c: class; o: order; f: family.

Supplementary Table 3. Reference protein sequences of the ratified proteins

Protein	Source	NCBI Accession	Reference
DsyB	*Labrenzia aggregata* IAM 12614	EAV42226	Curson et al., 2017
	Labrenzia aggregate LZB033	WP_075282486.1	Curson et al., 2017
	Pseduooceanicola batsensis HTCC2597	EAQ04968	Curson et al., 2017
	Pelagibaca bermudensis HTCC2601	EAU45958	Curson et al., 2017
	Amorphus coralli DSM 19760	WP_018697905	Curson et al., 2017
	Sediminimonas qiaohouensis DSM 21189	WP_018697905	Curson et al., 2017
	Sagittula stellate E37	WP_005854984.1	Curson et al., 2017
	Thalassobaculum salexigens DSM 19539	WP_084618911.1	Curson et al., 2017
MmtN	*Thalassospira profundimaris* PB8B	OAZ15224	Seebeck et al., 2019
	Novosphingobium sp. MBES04	GAM03459	Williams et al., 2019
	Roseovarius indicus B108	KRS18724	Williams et al., 2019
	Nocardiopsis chromatogenes YIM 90109	WP_017624909	Williams et al., 2019
	Streptomyces mobaraensis DSM 40847	EME99407	Williams et al., 2019
DSYB	*Prymnesium parvum* CCAP946/6	NA	Curson et al., 2018
	Chrysochromulina tobin CCMP291	KOO32714	Curson et al., 2018
	Lingulodinium polyedrum CCMP1936	NA	Curson et al., 2018
	Alexandrium tamarense ATSP1-B	NA	Curson et al., 2018
	Acropora cervicornis	NA	Curson et al., 2018
	Fragilariopsis cylindrus CCMP1102	OEU17621	Curson et al., 2018
	Symbiodinium microadriaticum CCMP2467	OLQ07620	Curson et al., 2018
DmdA	*Ruegeria pomeroyi* DSS-3	AAV95190	Howard et al., 2006
	Pelagibacter ubique HTCC1062	WP_011281570	Howard et al., 2006
	Dinoroseobacter shibae DFL 12	WP_012178987	Howard et al., 2008
	marine gammaproteobacterium HTCC2080	WP_007233625	Howard et al., 2008
	Candidatus Pelagibacter sp. HTCC7211	WP_008546106	Howard et al., 2010
	Candidatus Puniceispirillum marinum IMCC1322	WP_013044947	Curson et al., 2011b
DmdB	*Ruegeria pomeroyi*	WP_011047771.1	Reisch et al., 2011
	Ruegeria pomeroyi	WP_011046428.1	Reisch et al., 2011
	Candidatus Pelagibacter ubique	WP_011281571.1	Reisch et al., 2011

DmdC	*Ruegeria pomeroyi*	WP_011049476.1	Reisch et al., 2011
	Burkholderia thailandensis E264	WP_009892931.1	Reisch et al., 2011
	Ruegeria lacuscaerulensis ITI-1157	EEX10128.1	Reisch et al., 2011
	Ruegeria pomeroyi DSS-3	WP_011048615.1	Reisch et al., 2011
	Pseudomonas aeruginosa PAO1	WP_003114720.1	Reisch et al., 2011
	Pseudomonas aeruginosa PAO1	WP_003114561.1	Reisch et al., 2011
	Burkholderia thailandensis E264	WP_009889880.1	Reisch et al., 2011
	Ruegeria lacuscaerulensis ITI-1157	EEX08676.1	Reisch et al., 2011
DmdD	*Ruegeria pomeroyi*	Q5LLW6.1	Reisch et al., 2011<br>Tan et al., 2013
AcuH	*Ruegeria lacuscaerulensis* ITI-1157	EEX08788.1	Cao et al., 2017
	Ruegeria pomeroyi DSS-3	AAV93475.1	Cao et al., 2017
DddD	*Marinomonas* sp.	WP_012071702.1	Todd et al., 2007
	Oceanimonas doudoroffii	WP_094199898.1	Curson et al., 2012
	Psychrobacter sp. J466	ACY02894	Curson et al., 2010
	Halomonas sp.	ACV84065	Todd et al., 2010
	Burkholderia ambifaria	WP_011659284	Curson et al., 2010
	Pseudomonas sp. J465	ACY01992	Todd et al., 2007
DddL	*Sulfitobacter* sp. EE-36	ADK55772	Curson et al., 2008
	Rhodobacter sphaeroides 2.4.1	YP_351475	Curson et al., 2008
	Labrenzia aggregata IAM12614	WP_006936138.1	Curson et al., 2017
	Fulvimarina pelagi	WP_007067665.1	Curson et al., 2008
	Pseudooceanicola batsensis	WP_009805827.1	Curson et al., 2008
	Ahrensia marina LZD062	KP639183	Liu et al., 2018
DddP	*Roseovarius nubinhibens*	EAP77700	Kirkwood et al., 2010
	Ruegeria pomeroyi DSS-3	WP_044029245.1	Todd et al., 2011
	Fusarium graminearum PH-1	XP_389272	Todd et al., 2009
	Oceanimonas P1	WP_094198963.1	Curson et al., 2012
	Oceanimonas P2	WP_094200218.1	Curson et al., 2012
DddQ	*Ruegeria pomeroyi* DSS-3	WP_011047333	Todd et al., 2011
	Roseovarius nubinhibens 1	WP_009814827.1	Todd et al., 2011
	Roseovarius nubinhibens 2	WP_009814826.1	Todd et al., 2011
	Ruegeria lacuscaerulensis	WP_005978225	Li et al., 2014
	GOS databases 1	ECW91654	Todd et al., 2011
	GOS databases 2	EBP74803	Todd et al., 2011

	GOS databases 3	ECX82089	Todd et al., 2011
DddW	*Ruegeria pomeroyi* DSS-3	AAV93771	Todd et al., 2012
DddY	*Alcaligenes faecalis* M3A	ADT64689	Curson et al., 2012
	Shewanella putrefaciens CN-32	ABP77243	Curson et al., 2012
DddK	*Candidatus* Pelagibacter ubique HTCC1062	WP_011281678.1	Schnicker et al., 2017
	Alphaproteobacterium HIMB5	WP_014953073.1	Schnicker et al., 2017
	Candidatus Pelagibacter ubique HTCC1002	WP_006997514.1	Schnicker et al., 2017
	Pelagibacteraceae bacterium BACL20	KRP06000.1	Schnicker et al., 2017
	Candidatus Pelagibacter ubique 4	WP_027306832.1	Schnicker et al., 2017
	Candidatus Pelagibacter ubique 2	WP_018413735.1	Schnicker et al., 2017
	Candidatus Pelagibacter ubique 3	WP_028037226.1	Schnicker et al., 2017
Alma1	*Emiliania huxleyi* CCMP1516	XP_005784450	Alcolombri et al., 2015
	Emiliania huxleyi CCMP1516	XP_005763983	Alcolombri et al., 2015
	Symbiodinium sp. *clade D*	P0DN22	Alcolombri et al., 2015
MddA	*Mycobacterium tuberculosis* H37Rv	WP_003416945.1	Carrión et al., 2015
	Bradyrhizobium diazoefficiens USDA 110	WP_011084036.1	Carrión et al., 2015
	Bradyrhizobium diazoefficiens USDA 110	WP_011088485.1	Carrión et al., 2015
	Pseudomonas sp. GM41	WP_008148420.1	Carrión et al., 2015
	Pseudomonas deceptionensis	WP_048359798.1	Carrión et al., 2015
	Sulfurovum sp. NBC37-1	WP_011980608.1	Carrión et al., 2015
DmoA	*Hyphomicrobium sulfonivorans* 6AK1_A	NA	Boden et al., 2011
MTO	*Hyphomicrobium* sp.	ATJ26742.1	Lee et al., 2002
	Methylophaga thiooxydans	WP_008290534.1	Eyice et al., 2018

	Ruegeria pomeroyi	WP_011242048.1	Eyice et al., 2018
DdhA	*Ruegeria pomeroyi* DSS-3	WP_011242048.1	McDevitt et al., 2002
	Rhodovulum sulfidophilum	WP_060833690.1	McDevitt et al., 2002
Tmm	*Methylocella silvestris* BL2	ACK52489.1	Lidbury et al., 2016
	Ruegeria pomeroyi DSS-3	AAV94838.1	Lidbury et al., 2016
	Roseovarius sp. 217	EAQ26624.1	Lidbury et al., 2016
DMSOR	*Rhodobacter capsulatus* 1DMR_A	NA	Satoh and Kurihara., 1987
	Escherichia coli	WP_097479356.1	Sambasivarao and Weiner., 1991
	Rhodobacter sphaeroides	AAB94874.1	Mcewan et al., 1991

NA: not available. The protein sequences were as follows.

DSYB:

>Prymnesium_parvum_CCAP_946/6

MLRLAPRLPTRALVRHALRAHALPLARPALPSGTRLFASAPADDIDVDNVAYGFMASQALFTGLE
MGLFDAIAAGPEAGLNIDELKAAANCSAPRLQTLVTSLVAIKSLKRTPDGRYTLSPNTARFLVQSSK
QYYGDYLKYQMGRQFYHRMGALPDVMTSGEAPSYASWFSDPETAATYTQAQHNGSVATAKYLI
KKKLQLGDAATMLDVGGGSGAFSYVFTEATPGLSSTVLELPEVCRTGEAIKAKQPVSVQERVKLV
ELDATSPDWPVNDAAYDVVLMSYISGSVPESIIGALYANAYKALKPGGRLLVHDFMVDNSLDGPP
LGALWALQHVTVNADGLGLCPQGVIERMGSAGFDPSACETMEMITGLTKLIVAYKP

>Lingulodinium_polyedrum_CCMP1936

MAFAPRTSPLLARAVTRAWPRAQRSAAAALRSWARPALAAKPPLASRAFARTSWDEGEDVDLDS
VAYGFMASQALFTGLELGIFDHIAAAGAGGLSAAGIGKACGIEAPRVQTLLTSLVAVKCLKRDASA
MYTLSPNTAQYMVTSSRHFYGDYLRYQIGRQFYHRMGALPEVMTSGKAPSYASWFSDPEVARTY
TQAQHNGSVATAKYLIKKKLQLGGISAMLDVGGGSGAFSYVFTQATPGLHSKVLELPEVCRTGEGI
RAKQPEDVRSRVSFVELDASSPTWPVDDSAFDVVLMSYISGSVPEPIIGSLYANAMKALRPGGRLL
VHDFMVNDSLDGPALGALWGLQHVTVNADGLGLCPKEIISRMGTAGFDTSKCETMEMIHGMTKL
IVGYKN

>Alexandrium_tamarense_ATSP1B_1

MPPSRAWRRLAVSAQQILPSIVQGTSSEDVDLDALAYGFMASQALFSALELGIFDHLAACPATAPEL
GSACGIPTQRLQTLLTALVASRCLRLDAESLKYTNSPNVARFMVSNSKSYYGDYLKCQIGWLFYHR
MGRITEVMKGGEALDYQTWFSDPHVADTYTSAQHNGSLATARALMRKVDLSRVTCLLDAGGGS
GAFSIAAARAVPGLEATVLELPEVCKTGSRIVEQAGLSGRIRYVELDATSPDWPVHGGSFQAVLMS
YLSGSVPAHAIVGLYSNAYKALGPGGRLVVHDFMVDNSLDGPQLGALWALQHVTVNPDGLGLCP
GHVVSRMRTAGFARTETLDLIGGMTKVVVGYKD

>Acropora_cervicornis

MAAAFAAARGLTRAISRCASKRVPEQALFSRLSAPAPPALAALPGVRAFSRTSWESGDDVDLDSVA
YGFMASQALFSALELGIFDKVAAAGEKGCAAKDVQQACGVEGPRLTTLLTALTAVKCLRRSDEGL
YTLSPNTAQYMVSSSRHYYGDYLQYQIGRQFYHRMGALPEVMTTGKAPSYASWFSDPEVAKTYT

QAQHNGSVATAKYLVRKKLDLGGISSMLDVGGGSGAFSYVFTEATPGLKSTVLELPEVCRTGEGIK
AQQPQDIQDRVSFVELDATSPDWPVSDSNYDIVLMSYISGSVPESVILPLYKNAFKALRPGGRLLVH
DFMVNDSLDGPALGALWGLQHVTVNAQGLGLCPAEVIRRMAQAGFEENKCQTHEMIHGMTKLI
VAHKA

DmoA:
> Hyphomicrobium_sulfonivorans_6AK1_A
MKKRIVLNAFDMTCVSHQSAGTWRHPSSQAARYNDLEYWTNMAMELERGCFDCLFIADVVGVY
DVYRGSAEMALRDADQVPVNDPFGAISAMAAVTEHVGFGVTAAITFEQPYLLARRLSTLDHLTKG
RVAWNVVSSYLNSAALNIGMDQQLAHDERYEMADEYMEVMYKLWEGSWEDDAVKRDKKSGVF
TDGSKVHPINHQGKYYKVPGFHICEPSPQRTPVIFQAGASGRGSKFAASNAEGMFILTTSVEQARQI
TTDIRNQAEAAGRSRDSIKIFMLLTVITGDSDEAAEAKYQEYLSYANPEGMLALYGGWTGIDFAKL
DPDEPLQAMENDSLRTTLESLTHGENAKKWTVRDVIRERCIGGLGPVLVGGPQKVADELERWVDE
GGVDGFNLAYAVTPGSVTDFIDYIVPELRKRGRAQDSYKPGSLRRKLIGTNDGRVESTHPAAQYRD
AYVGKESVADRTQPSPFANAKAPVAE

DMSOR
> Rhodobacter Capsulatus 1DMR_A
MTKFSGNELRAELYRRAFLSYSVAPGALGMFGRSLLAKGARAEALANGTVMSGSHWGVFTATVENGR
ATAFTPWEKDPHPSPMLAGVLDSIYSPTRIKYPMVRREFLEKGVNADRSTRGNGDFVRVSWDQALDLV
AAEVKRVEETYGPEGVFGGSYGWKSPGRLHNCTTLLRRMLTLAGGYVNGAGDYSTGAAQVIMPHVV
GTLEVYEQQTAWPVLAENTEVMVFWAADPIKTSQIGWVIPEHGAYPGLEALKAKGTKVIVIDPVRTKT
VEFFGAEHITPKPQTDVAIMLGMAHTLVAEDLYDKDFIANYTSGFDKFLPYLDGETDSTPKTAEWAEGIS
GVPAETIKELARLFESKRTMLAAGWSMQRMHHGEQAHWMLVTLASMLGQIGLPGGGFGLSYHYSGG
GTPSTSGPALAGITDGGAATKGPEWLAASGASVIPVARVVDMLENPGAEFDFNGTRSKFPDVKMAYWV
GGNPFVHHQDRNRMVKAWEKLETFVVHDFQWTPTARHADIVLPATTSYERNDIETIGDYSNTGILAMK
KIVEPLYEARSDYDIFAAVAERLGKGAEFTEGKDEMGWIKSFYDDAAKQGKAAGVQMPAFDAFWAEGI
VEFPVTDGADFVRYASFREDPLLNPLGTPTGLIEIYSKNIEKMGYDDCPAHPTWMEPLERLDGPGAKYP
LHIAASHPFNRLHSQLNGTVLREGYAVQGHEPCLMHPDDAAARGIADGDVVRVHNDRGQILTGVKVT
DAVMKGVIQIYEGGWYDPSDVTEPGTLDKYGDVNVLSADIGTSKLAQGNCGQTVLAEVEKYTGPAVT
LTGFVAP

Supplementary Table 4. The information of key proteins involved in cycling DMSP and related compounds.

Enzyme	Polypeptide class	Reaction catalysed	E-value used in this study	Reference
DddP	M24B metallopeptidase family	DMSP cleavage to DMS and acrylate, plus a proton	E ⩽ 1×10^{-83}	Todd et al., 2009<br>Curson et al., 2011<br>Lei et al., 2018
DddQ	Cupin superfamily	DMSP cleavage to DMS and acrylate, plus a proton	E < 1×10^{-20}	Todd et al., 2011<br>Curson et al., 2011<br>Lei et al., 2018
DddW	Cupin superfamily	DMSP cleavage to DMS and acrylate, plus a proton	E < 1×10^{-49}	Todd et al., 2012<br>Curson et al., 2011<br>Lei et al., 2018
DddY	Cupin superfamily	DMSP cleavage to DMS and acrylate, plus a proton	E ⩽ 1×10^{-64}	Curson et al., 2011a<br>Li et al., 2017<br>Lei et al., 2018
DddL	Cupin superfamily	DMSP cleavage to DMS and acrylate, plus a proton	E ⩽ 1×10^{-33}	Curson et al., 2008<br>Lei et al., 2018<br>Curson et al., 2011
DddK	Cupin superfamily	DMSP cleavage to DMS and acrylate, plus a proton	E ⩽ 1×10^{-35}	Schnicker et al., 2017<br>Lei et al., 2018
DddD	Class III CoA transferase family	DMSP cleavage to DMS and 3HP, probably involving a DMSP-CoA intermediate	E ⩽ 1×10^{-97}	Todd et al., 2007<br>Curson et al., 2011<br>Lei et al., 2018
Alma1 (eukaryotic)	Aspartic acid/glutamic acid/ethylurea racemase superfamily	DMSP cleavage to DMS and acrylate	E ⩽ 1×10^{-26}	Alcolombri et al., 2015<br>Lei et al., 2018
DmdA	Glycine cleavage system T family	DMSP demethylation to MMPA	E ⩽ 1×10^{-130}	Reisch et al., 2008<br>Curson et al., 2011b<br>González et al., 2019
DmdB	AMP combinase superfamily	Catalyze MMPA to MMPA-CoA	E ⩽ 1×10^{-75}	Reisch et al., 2011
DmdC	Acyl CoA dehydrogenase superfamily	MMPA-CoA dehydrogenation to MTA-CoA	E ⩽ 1×10^{-100}	Reisch et al., 2011
DmdD	Allyl hydrase superfamily	MTA-CoA hydration to acetaldehyde	E < 1×10^{-30}	Reisch et al., 2011<br>Tan et al., 2013
AcuH	Allyl hydrase superfamily	MTA-CoA hydration to acetaldehyde	E ⩽ 1×10^{-56}	Cao et al., 2017

DsyB	SAM-dependent methyltransferase	MTHB methylation to DMSHB	E ≤ 1×10^{-67}	Curson et al., 2017
MmtN (MSMT)	Met-methylating enzymes	Methionine methylation to SMM	E ≤ 1×10^{-98}	Seebeck et al., 2019<br>Williams et al., 2019
DSYB (eukaryotic)	SAM-dependent methyltransferase	MTHB methylation to DMSHB	E ≤ 1×10^{-30}	Curson et al., 2018
MddA	Methanethiol S-methyltransferase	Catalyzes MeSH to DMS	E < 1×10^{-30}	Carrión et al., 2015
DmoA	Dimethylsulfide monooxgenase	DMS assimilation to MeSH	E ≤ 1×10^{-34}	Boden et al., 2011
MTO	Methanethiol oxidase	MeSH oxidation to Formaldehyde	phylogenetic tree and re-blast	Lee et al., 2002<br>Eyice et al., 2018
DdhA	Dimethylsulfide dehydrogenase	DMS oxidation to DMSO	phylogenetic tree and re-blast	McDevitt et al., 2002
Tmm	Trimethylamine monooxygenase	DMS oxidation	phylogenetic tree and re-blast	Lidbury et al., 2016
DMSOR	Dimethyl sulphoxide reductase	DMSO reduction to DMS	E ≤ 1×10^{-94}	Satoh and Kurihara., 1987<br>Sambasivarao and Weiner., 1991<br>Mcewan et al., 1991

Note: 3HP, 3-hydroxypropionate; DMS, dimethyl sulfide; DMSP, dimethylsulfoniopropionate; MMPA, methylmercaptopropionate; MTHB, 4-methylthio-2-hydroxybutyrate; DMSHB, 4-dimethylsulfonio-2-hydroxybutyrate; SMM, S-methylmethionine. MeSH, methanethiol; MTA-CoA, methylthioacryloyl-CoA.

Supplementary Table 5. The numbers of gene homologues in metagenomes from different samples.

Station	*dsyB*	*mmtN*	*DSYB*	*Alma1*	*dddD*	*dddK*	*dddL*	*dddP*	*dddQ*	*dmdA*	*dmdB*	*dmdC*	*dmdD*	*AcuH*	*mddA*	*dmoA*	*ddhA*	*mtoX*	*tmm*	*DMSOR*	*recA*	*β-Actin*
H12m	9	NT	NT	NT	43	NT	NT	712	94	212	796	841	43	375	211	33	10	NT	NT	44	1593	296
H12sw	24	NT	6	512	49	275	1	520	471	1562	955	1525	59	296	21	74	NT	9	4	149	3331	5537
H12bw	23	NT	1	NT	2	269	11	596	297	1798	2218	1616	133	384	12	169	67	29	26	320	4929	502
HS5m	34	NT	NT	NT	112	NT	13	827	99	172	1157	939	69	451	324	69	89	7	20	52	2180	402
HS5sw	16	NT	38	1149	35	80	NT	151	241	1703	1240	1649	72	343	6	42	NT	8	8	214	4042	6968
HS5bw	25	NT	10	175	9	282	7	643	360	2151	3026	2143	136	553	39	103	3	37	37	385	6214	2577
H25	22	NT	NT	NT	49	NT	85	611	54	139	1106	779	2	247	171	10	12	22	19	27	2084	1
B41	21	NT	NT	2	59	NT	36	527	78	128	801	589	0	258	194	8	29	13	19	31	1560	93
B45	37	NT	NT	2	81	NT	20	970	143	297	1137	898	0	382	256	14	24	5	52	52	2262	218
B47	16	NT	NT	NT	73	NT	43	637	31	89	553	429	0	317	116	5	6	45	14	28	1404	125
B50	34	NT	NT	NT	64	NT	47	816	120	239	1395	936	7	380	216	14	23	16	29	61	2574	1
T1	1	NT	NT	NT	NT	NT	NT	NT	NT	NT	34	75	0	39	21	NT	NT	5	NT	NT	293	NT
TVG11-2	8	NT	NT	NT	NT	NT	NT	NT	NT	NT	137	109	7	57	19	15	68	NT	NT	23	1547	NT
TVG5-3	NT	NT	NT	NT	NT	NT	NT	NT	NT	NT	225	248	7	148	44	36	85	NT	NT	3	1276	NT
TVGS11-2	NT	NT	NT	NT	NT	NT	NT	NT	NT	NT	63	5	6	18	1	5	1	NT	NT	NT	1723	NT
TVGS5-3	29	NT	NT	NT	NT	NT	NT	1	NT	NT	34	202	3	137	4	6	72	NT	NT	11	1508	NT

Note: m, sediment; sw, surface seawater; bw, bottom seawater; TVG, sediment; TVGS, polymetallic sulfide; NT, not detected.

Supplementary Figures

$$G_k = \frac{r_k}{L_k} \cdot \frac{1}{\sum_{i=1}^{n} \frac{r_i}{L_i}}$$

Supplementary Figure 1. Metagenomics non-redundant gene set (Unigenes/protein) abundance calculation method. Gk represents the relative abundance of the gene k in the sample, rk is the number of times the gene k is detected in the sample, equal to the number of aligned reads, Lk is the length of gene k, the total number of ri samples and the total length of Li samples (Villar et al., 2015).

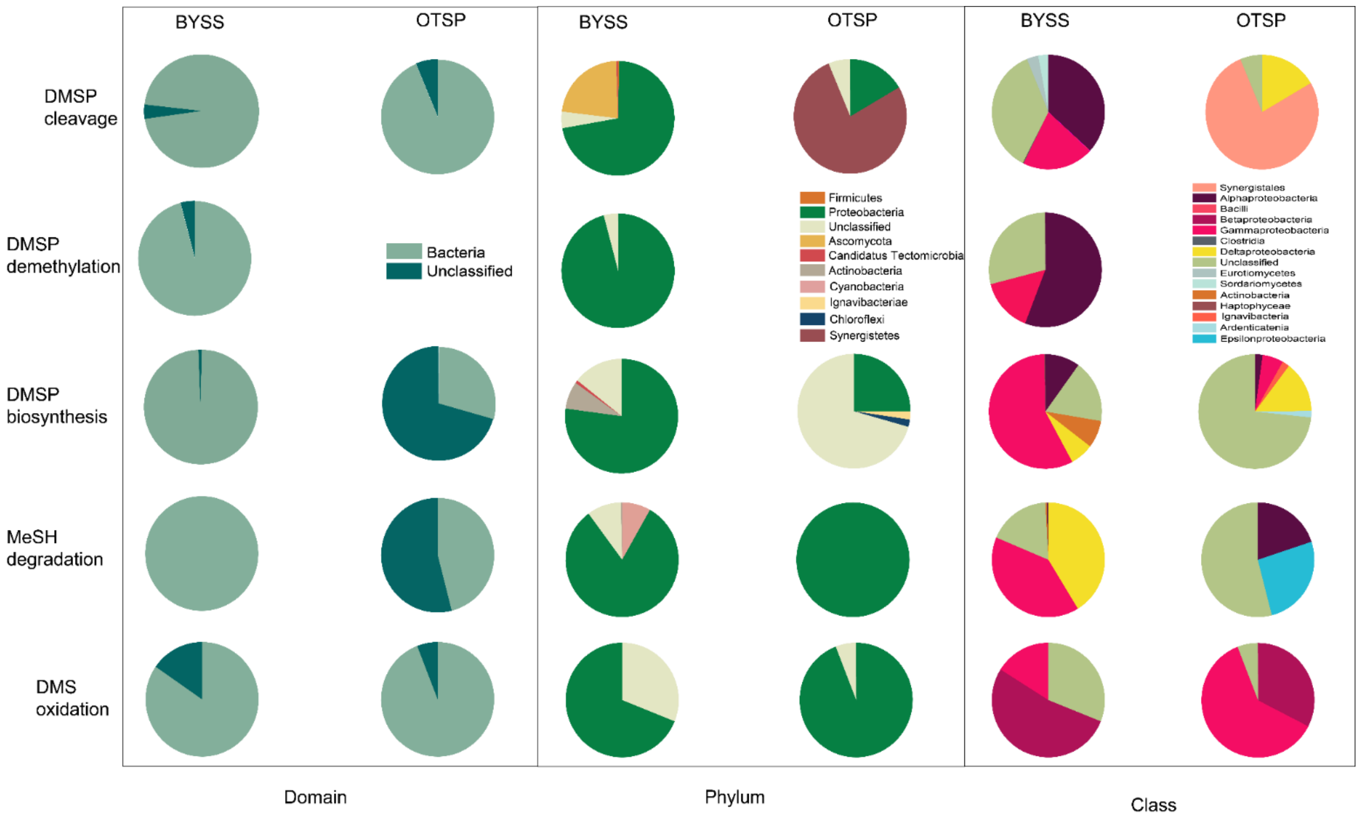

Supplementary Figure 2. The taxa implicated in the production and cycling of DMSP and related compounds at Domain, Phylum and Class taxonomic levels in metagenomics data from eathern Chinese marginal sea sediment samples. BYSS, sediment of the Yellow Sea and the Bohai Sea; OTSP, sediment and polymetallic sulfide of the Okinawa Trough.

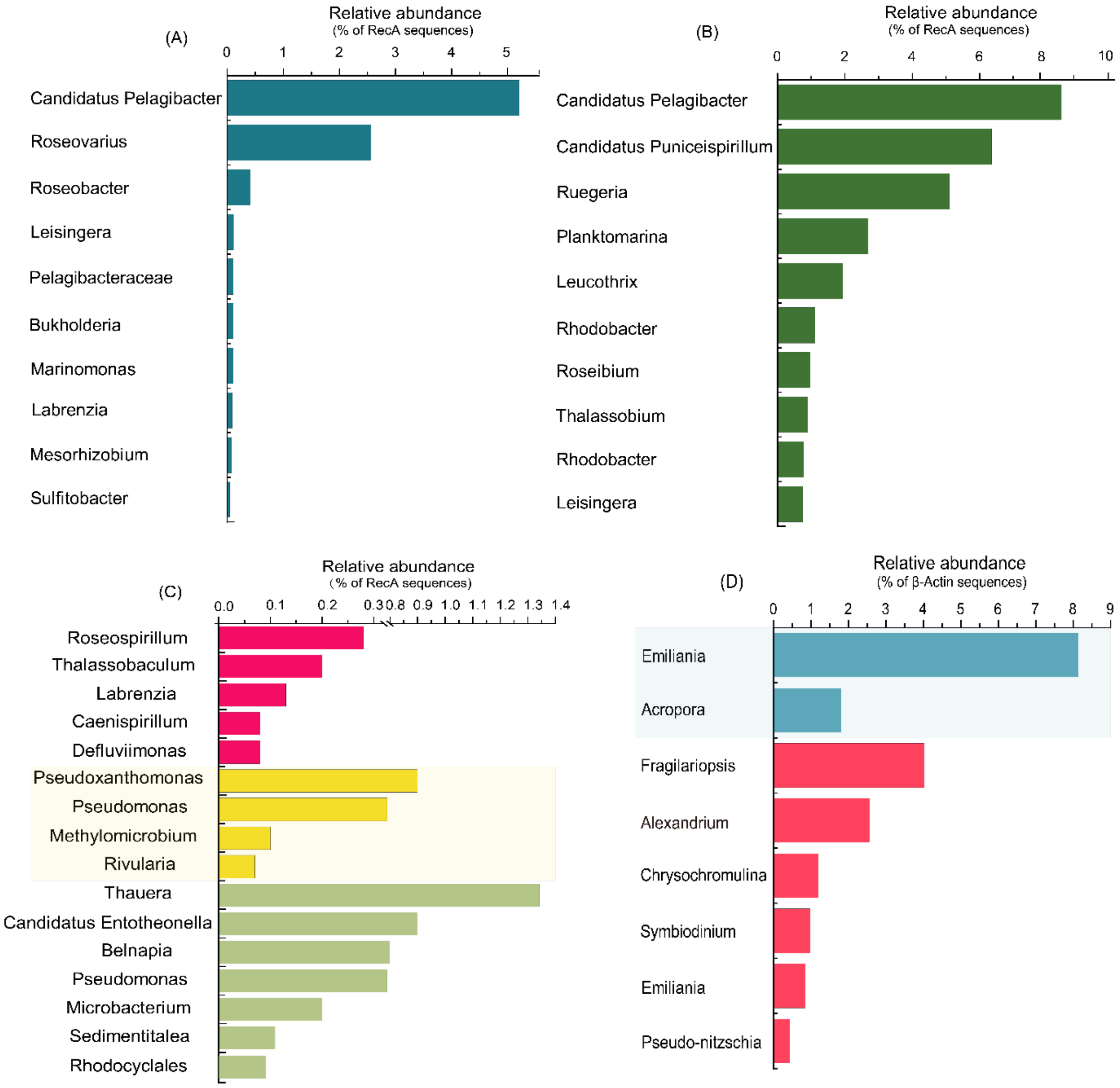

Supplementary Figure 3. The most abundant genera involved in the production and cycling of DMSP and related compounds in the Yellow Sea seawater samples (sites H12 and HS5). A: The most abundant genera possessing "*ddd*" genes; B: The most abundant genera possessing *dmdA*; C: The most abundant genera possessing *dsyB* with red, the most abundant genera of MeSH degradation with yellow and the most abundant genera of DMS oxidation with green; D: The most abundant genera possessing *Alma1* with blue, The most abundant genera possessing *DSYB* with red.

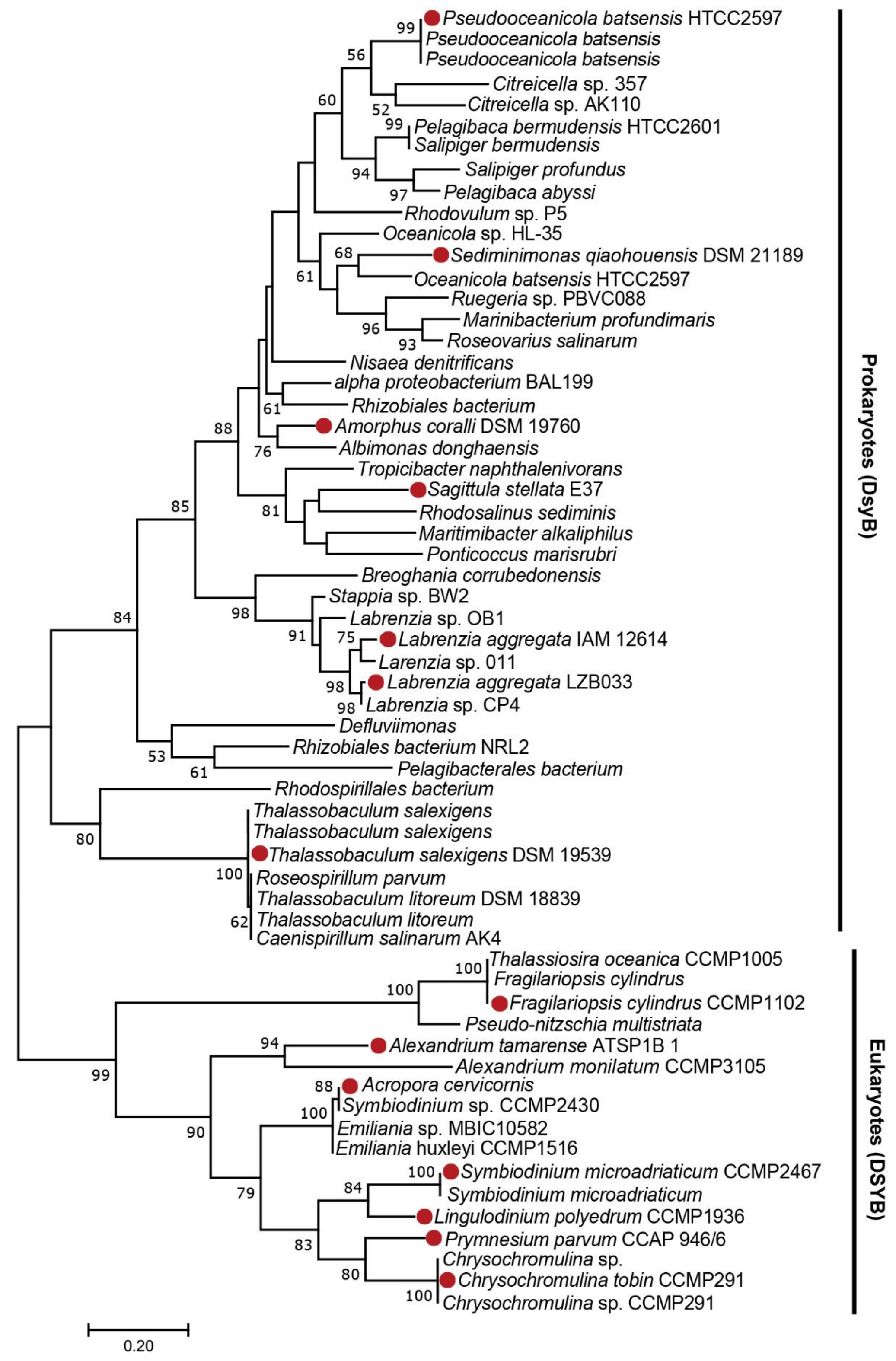

Supplementary Figure 4. Maximum likelihood phylogenetic tree of DsyB and DSYB proteins. Ratified proteins of DsyB and DSYB (Supplementary Material Table 3) available from NCBI alongside the newly retrieved sequences from metagenomes were used for phylogenetic tree reconstruction. The ratified proteins were marked with a red solid circle. Bootstrap support for nodes is marked.

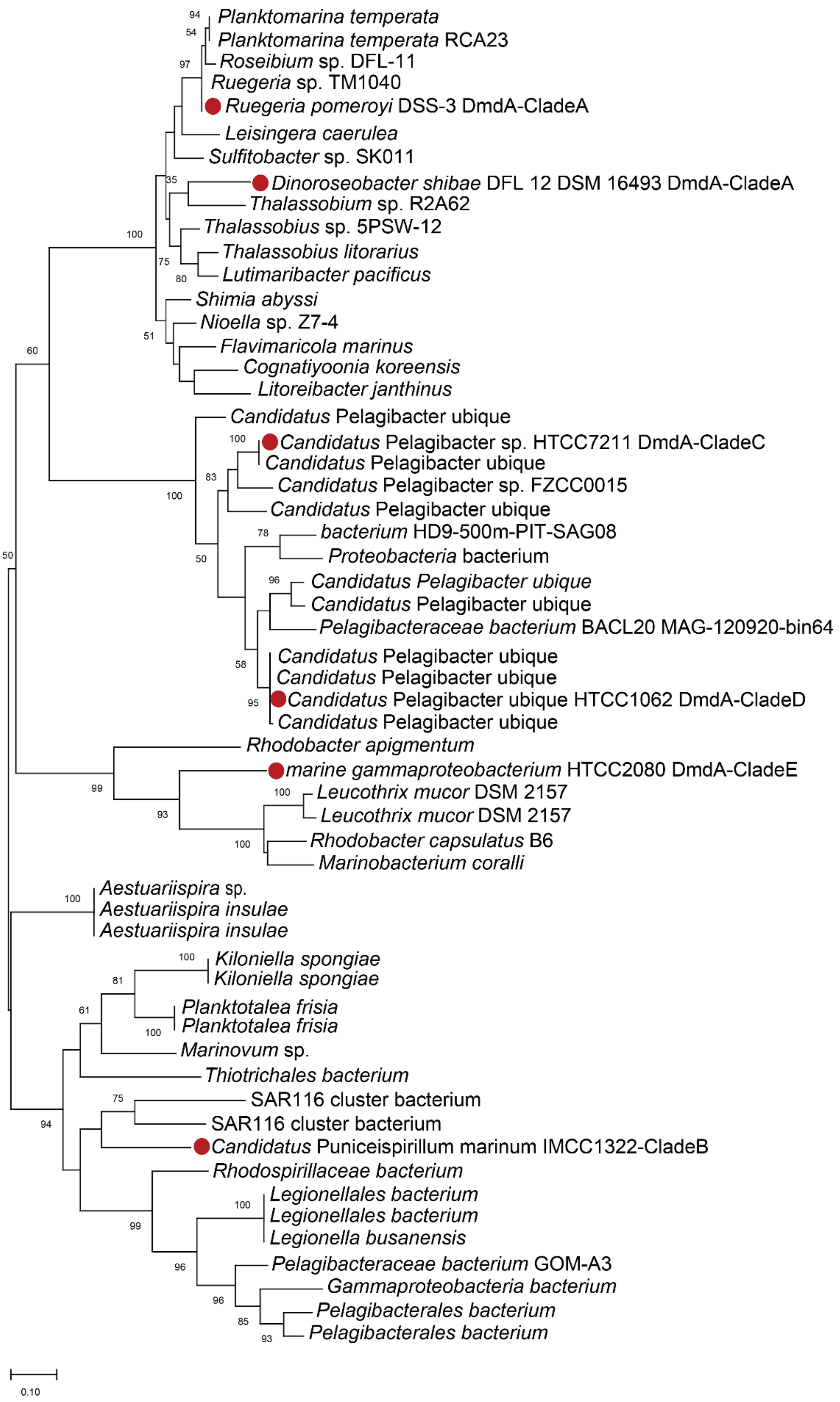

Supplementary Figure 5. Maximum likelihood phylogenetic tree of DmdA proteins. Ratified proteins of DmdA (Supplementary Material Table 3) available from NCBI alongside the newly retrieved sequences from metagenomes were used for phylogenetic tree reconstruction. The ratified proteins were marked with a red solid circle. Bootstrap support for nodes is marked.

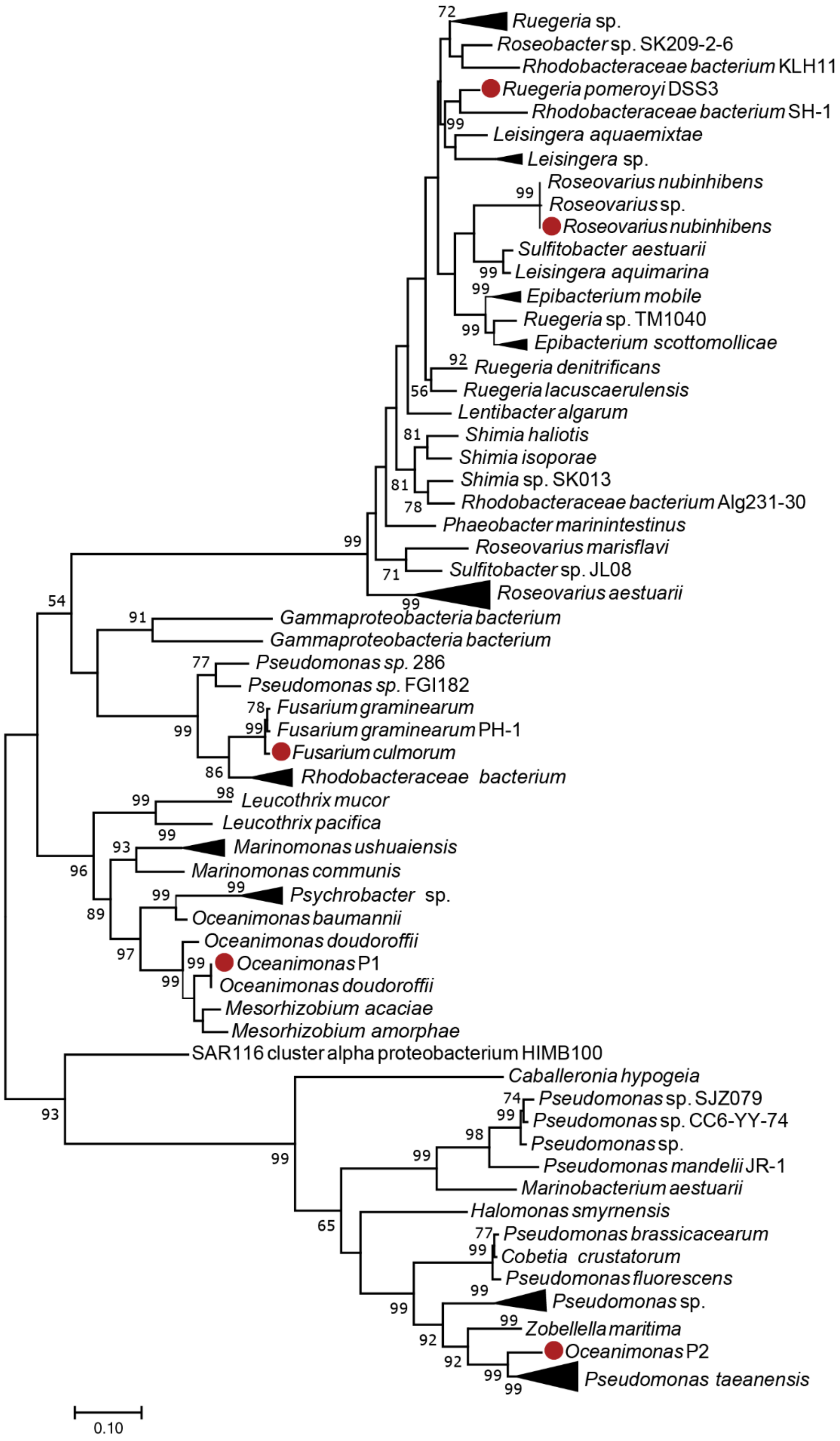

Supplementary Figure 6. Maximum likelihood phylogenetic tree of DddP proteins. Ratified proteins of DddP (Supplementary Material Table 3) available from NCBI alongside the newly retrieved sequences from metagenomes were used for phylogenetic tree reconstruction. The ratified proteins were marked with a red solid circle. Bootstrap support for nodes is marked.

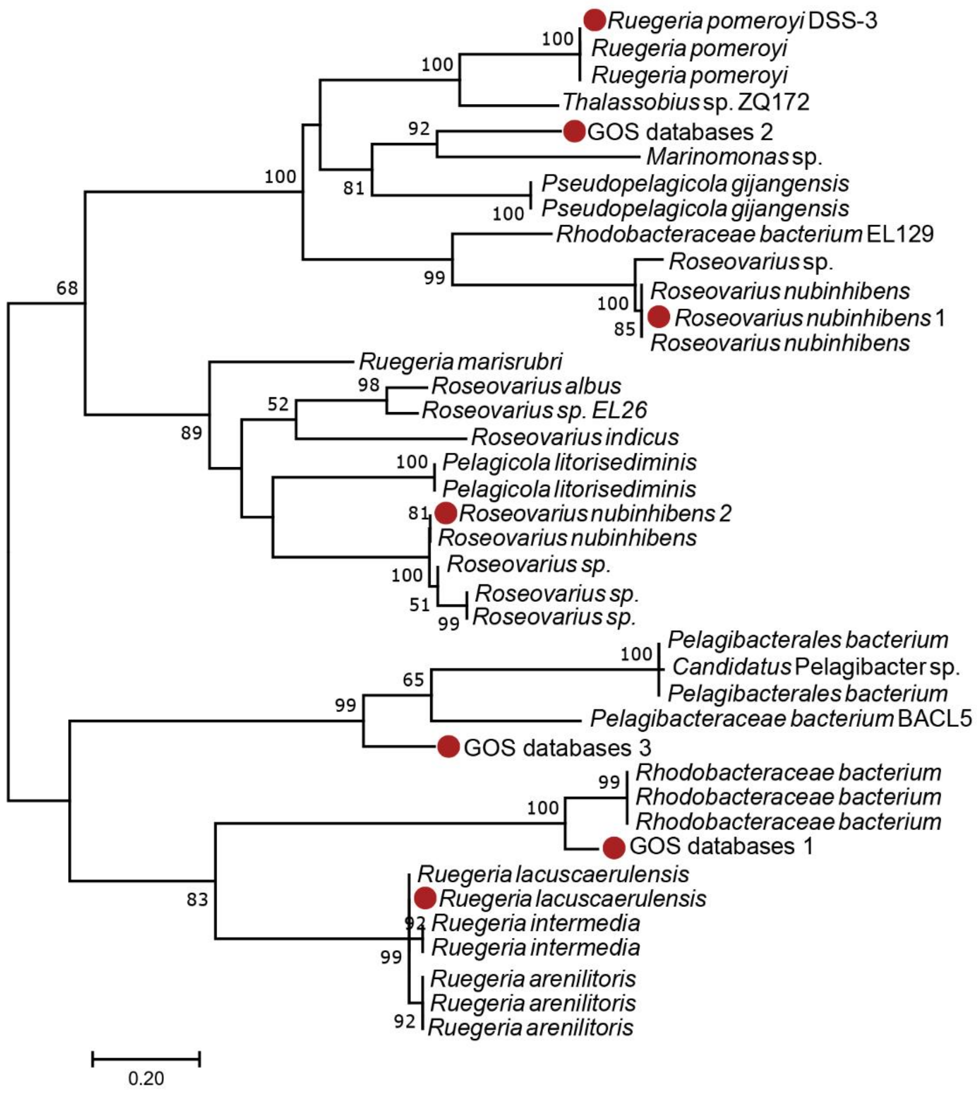

Supplementary Figure 7. Maximum likelihood phylogenetic tree of DddQ proteins. Ratified proteins of DddQ (Supplementary Material Table 3) available from NCBI alongside the newly retrieved sequences from metagenomes were used for phylogenetic tree reconstruction. The ratified proteins were marked with a red solid circle. Bootstrap support for nodes is marked.

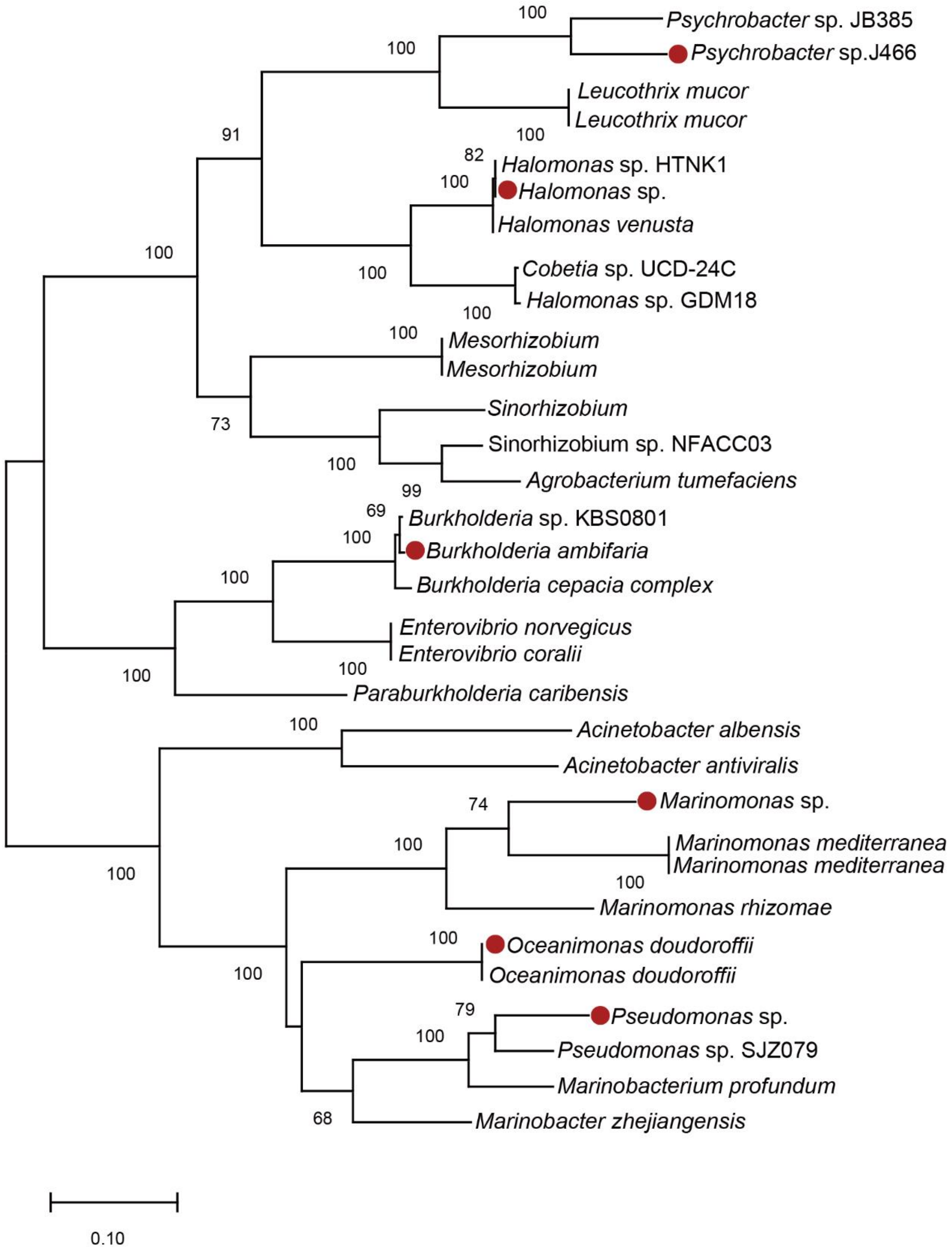

Supplementary Figure 8. Maximum likelihood phylogenetic tree of DddD proteins. Ratified proteins of DddD (Supplementary Material Table 3) available from NCBI alongside the newly retrieved sequences from metagenomes were used for phylogenetic tree reconstruction. The ratified proteins were marked with a red solid circle. Bootstrap support for nodes is marked.

0

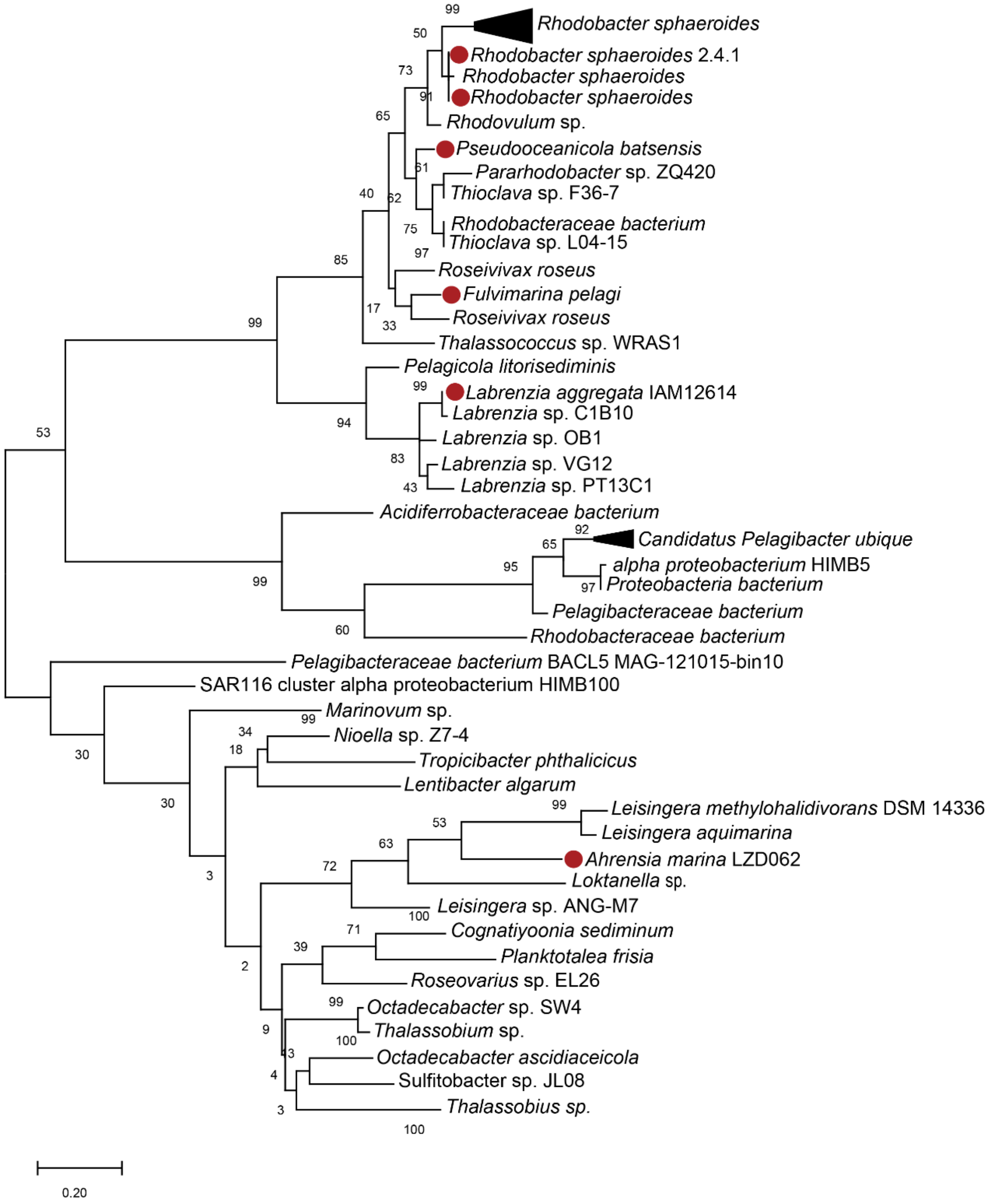

Supplementary Figure 9. Maximum likelihood phylogenetic tree of DddL proteins. Ratified proteins of DddL (Supplementary Material Table 3) available from NCBI alongside the newly retrieved sequences from metagenomes were used for phylogenetic tree reconstruction. The ratified proteins were marked with a red solid circle. Bootstrap support for nodes is marked.

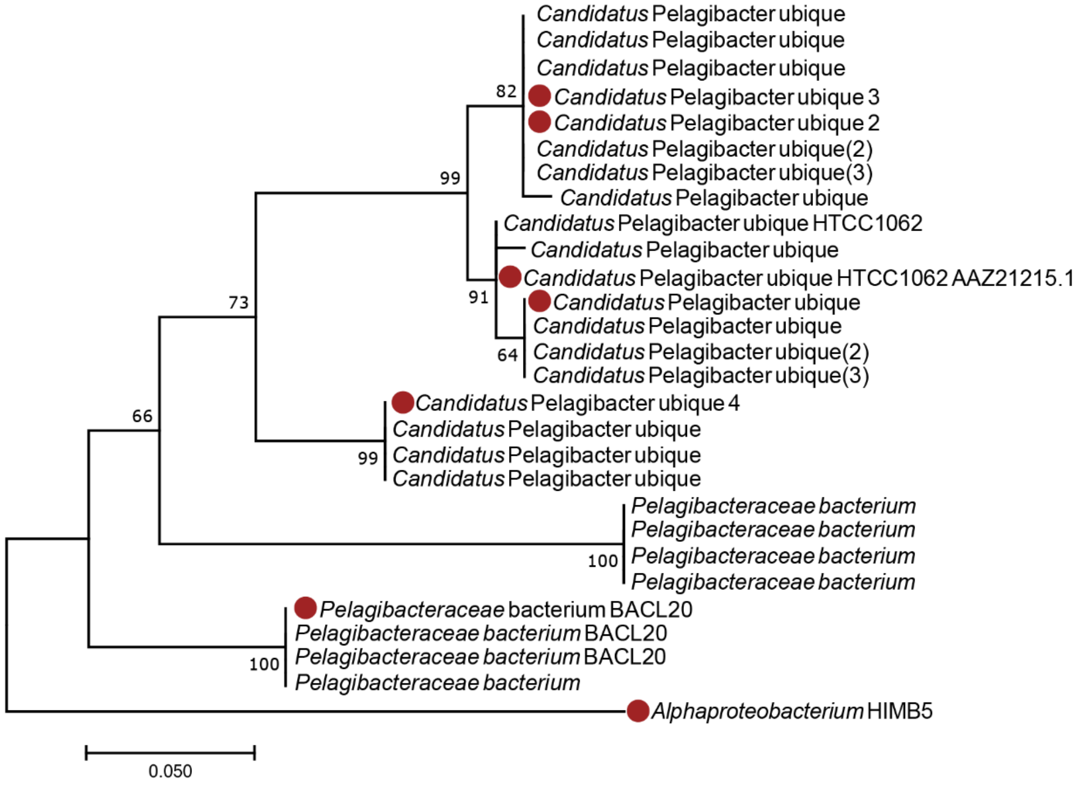

Supplementary Figure 10. Maximum likelihood phylogenetic tree of DddK proteins. Ratified proteins of DddK (Supplementary Material Table 3) available from NCBI alongside the newly retrieved sequences from metagenomes were used for phylogenetic tree reconstruction. The ratified proteins were marked with a red solid circle. Bootstrap support for nodes is marked.

References for supplementary materials

Alcolombri, U., Ben-Dor, S., Feldmesser, E., Levin, Y., Tawfik, D. S., and Vardi, A. (2015). Identification of the algal dimethyl sulfide–releasing enzyme: a missing link in the marine sulfur cycle. *Science*. 348, 1466-1469. doi: 10.1126/science.aab1586.

Boden, R., Borodina, E., Wood, A. P., Kelly, D. P., Murrell, J. C., and Schäfer, H. (2011). Purification and characterization of dimethylsulfide monooxygenase from *Hyphomicrobium sulfonivorans*. *J. Bacteriol.* 193, 1250-1258. doi: 10.1128/JB.00977-10.

Cao, H. Y., Wang, P., Xu, F., Li, P. Y., Xie, B. B., and Qin, Q. L. (2017). Molecular insight into the acryloyl-CoA hydration by AcuH for acrylate detoxification in dimethylsulfoniopropionate-catabolizing abcteria. *Front. Microbiol*. 8 : 2034. doi: 10.3389/fmicb.2017.02034.

Carrión, O., Curson, A. R. J., Kumaresan, D., Fu, Y., Lang, A. S., and Mercadé, E. (2015). A novel pathway producing dimethylsulphide in bacteria is widespread in soil environments. *Nat. Commun*. 6: 6579. doi: 10.1038/ncomms7579.

Curson, A. R., Fowler, E. K., Dickens, S., Johnston, A. W., and Todd, J. D. (2012). Multiple DMSP lyases in the γ-proteobacterium Oceanimonas doudoroffii. *Biogeochemistry* 110, 109–119. doi:10.1007/s10533-011-9663-2.

Curson, A. R., Rogers, R., Todd, J. D., Brearley, C. A., and Johnston, A. W. (2008). Molecular genetic analysis of a dimethylsulfoniopropionate lyase that liberates the climate-changing gas dimethylsulfide in several marine α-proteobacteria and *Rhodobacter sphaeroides*. *Environ. Mi Jcrobiol*. 10, 757-767. doi: 10.1111/j.1462-2920.2007.01499.x.

Curson, A. R., Liu, J., Martínez, A. B., Green, R. T., Chan, Y., and Carrión, O. (2017). Dimethylsulfoniopropionate biosynthesis in marine bacteria and identification of the key gene in this process. *Nat. Microbiol*. 2: 17009. doi: 10.1038/nmicrobiol.2017.9.

Curson, A. R., Sullivan, M. J., Todd, J. D., and Johnston, A. W. (2011a). DddY, a periplasmic dimethylsulfoniopropionate lyase found in taxonomically diverse species of Proteobacteria. *ISME J*. 5: 1191. doi: 10.1038/ismej.2010.203.

Curson, A. R., Todd, J. D., Sullivan, M. J., and Johnston, A. W. (2011b). Catabolism of dimethylsulphoniopropionate: microorganisms, enzymes and genes. *Nat. Rev. Microbiol*. 9, 849. doi: 10.1038/nrmicro2653.

Curson, A. R., Sullivan, M. J., Todd, J. D., and Johnston, A. W. (2010). Identification of genes for dimethyl sulfide production in bacteria in the gut of Atlantic Herring (Clupea harengus). *ISME J.* 4, 144–146. doi:10.1038/ismej.2009.93.

Curson, A. R., Williams, B. T., Pinchbeck, B. J., Sims, L. P., Martínez, A. B., and Rivera, P. P. (2018). DSYB catalyses the key step of dimethylsulfoniopropionate biosynthesis in many phytoplankton. *Nat. Microbiol*. 3: 430. doi: 10.1038/s41564-018-0119-5.

Eyice, Myronova, N., Pol, A., Carrión, O., Todd, J. D., Smith, T. J., et al. (2018). Bacterial SBP56 identified as a Cu-dependent methanethiol oxidase widely

distributed in the biosphere. *ISME J*. 12, 145–160. doi: 10.1038/ismej.2017.148.
González, J. M., Hernández, L., Manzano, I., and Pedrós-Alió, C. (2019). Functional annotation of orthologs in metagenomes: a case study of genes for the transformation of oceanic dimethylsulfoniopropionate. *ISME J.* 13, 1183-1197. doi:10.1038/s41396-019-0347-6.
Goyer, A., Collakova, E., Shachar-Hill, Y., and Hanson, A. D. (2007). Functional characterization of a methionine γ-lyase in Arabidopsis and its implication in an alternative to the reverse trans-sulfuration pathway. *Plant. Cell. Physiol*. 48, 232-242. doi:10.1093/pcp/pcl055.
Howard, E. C., Sun, S., Biers, E. J., and Moran, M. A. (2008). Abundant and diverse bacteria involved in DMSP degradation in marine surface waters. *Environ. Microbiol.* 10, 2397–2410. doi:10.1111/j.1462-2920.2008.01665.x.
Howard, E. C., Henriksen, J. R., Buchan, A., Reisch, C. R., Bürgmann, H., and Welsh, R. (2006). Bacterial taxa that limit sulfur flux from the ocean. *Science* 314, 649-652. doi: 10.1126/science.1130657.
Howard, E. C., Sun, S., Reisch, C. R., del Valle, D. A., Bürgmann, H., and Kiene, R. P., et al. (2010). Changes in Dimethylsulfoniopropionate Demethylase Gene Assemblages in Response to an Induced Phytoplankton Bloom. *Appl. Environ. Microbiol.* 77, 524–531. doi:10.1128/aem.01457-10.
Kageyama, H., Tanaka, Y., Shibata, A., Waditee-Sirisattha, R., and Takabe, T. (2018). Dimethylsulfoniopropionate biosynthesis in a diatom *Thalassiosira pseudonana*: Identification of a gene encoding MTHB-methyltransferase. *Arch. Biochem. Biophys*. 645, 100-106. doi: 10.1016/j.abb.2018.03.019.
Kirkwood, M., Le Brun, N. E., Todd, J. D., and Johnston, A. W. (2010). The dddP gene of *Roseovarius nubinhibens* encodes a novel lyase that cleaves dimethylsulfoniopropionate into acrylate plus dimethyl sulfide. *Microbiology* 156, 1900–1906. doi:10.1099/mic.0.038927-0.
Lee, H. H., Kim, S. J., Shin, H.-J., Park, J. Y., and Yang, J. W. (2002). Purification and characterisation of methyl mercaptan oxidase from Thiobacillus thioparus for mercaptan detection. *Biotechnol. Bioprocess. Eng.* 7, 375–379. doi: 10.1007/bf02933525.
Lei, L., Cherukuri, K. P., Alcolombri, U., Meltzer, D., and Tawfik, D. S. (2018). The dimethylsulfoniopropionate (DMSP) lyase and lyase-like cupin family consists of bona fide DMSP lyases as well as other enzymes with unknown function. *Biochem*. 57, 3364-3377. doi: 10.1021/acs.biochem.8b00097.
Li, C. Y., Wei, T. D., Zhang, S. H., Chen, X. L., Gao, X., and Wang, P., et al. (2014). Molecular insight into bacterial cleavage of oceanic dimethylsulfoniopropionate into dimethyl sulfide. *Proc. Natl. Acad. Sci. U. S. A.* 111, 1026–31. doi:10.1073/pnas.1312354111.
Li, C. Y., Zhang, D., Chen, X. L., Wang, P., Shi, W. L., and Li, P. Y. (2017). Mechanistic insights into dimethylsulfoniopropionate lyase DddY, a new member of the cupin superfamily. *J. Mol. Biol.* 429, 3850-3862. doi: 10.1016/j.jmb.2017.10.022.
Lidbury, I., Kröber, E., Zhang, Z., Zhu, Y., Murrell, J. C., Chen, Y., et al. (2016). A

mechanism for bacterial transformations of DMS to DMSO: a missing link in the marine organic sulfur cycle. *Environ. Microbiol.* 18, 2754–2765. doi: 10.1111/1462-2920.13354.

Liu, J. L., Liu, J., Zhang, S. H., Liang, J., Lin, H., Song, D. L. et al. (2018). Novel insights into bacterial dimethylsulfoniopropionate catabolism in the East China Sea. *Front. Microbiol.* 9: 3206. doi: 10.3389/fmicb.2018.03206.

McDevitt, C. A., Hanson, G. R., Noble, C. J., Cheesman, M. R., and McEwan, A. G. (2002). Characterization of the redox centers in dimethyl sulfide dehydrogenase from *Rhodovulum sulfidophilum*. *Biochemistry* 41, 15234–15244. doi: 10.1021/bi026221u.

Mcewan, A. G.; Ferguson, S. J.; and Jackson, J. Baz. (1991). Purification and properties of dimethyl sulphoxide reductase from *Rhodobacter capsulatus*. A periplasmic molybdoenzyme. *Biochem. J.* 274, 305-307.

Reisch, C. R., Stoudemayer, M. J., Varaljay, V. A., Amster, I. J., Moran, M. A., and Whitman, W. B. (2011). Novel pathway for assimilation of dimethylsulphoniopropionate widespread in marine bacteria. *Nature* 473: 208. doi: 10.1038/nature10078.

Sambasivarao, D., and Weiner, J. H. (1991). Dimethyl sulfoxide reductase of Escherichia coli: an investigation of function and assembly by use of in vivo complementation. *J. Bacteriol.* 173, 5935–5943. doi:10.1128/jb.173.19.5935-5943.1991.

Satoh, T., and Kurihara, F. N. (1987). Purification and Properties of Dimethylsulfoxide Reductase Containing a Molybdenum Cofactor from a Photodenitrifier, *Rhodopseudomonas sphaeroides* f.s. *denitrificans*. *J. Biochem.* 102, 191–197. doi:10.1093/oxfordjournals.jbchem.a122.

Schnicker, N. J., De Silva, S. M., Todd, J. D., and Dey, M. (2017). Structural and biochemical insights into dimethylsulfoniopropionate cleavage by cofactor-bound DddK from the prolific marine bacterium *Pelagibacter*. *Biochemistry* 56, 2873-2885. doi: 10.1021/acs.biochem.7b00099.

Seebeck, F. P., and Liao, C. (2019). In vitro reconstitution of bacterial DMSP biosynthesis. *Angew. Chem. Int. Ed. Engl.* 131, 3591–3594. doi: 10.1002/ange.201814662.

Tan, D., Crabb, W. M., Whitman, W. B., and Tong, L. (2013). Crystal structure of DmdD, a crotonase superfamily enzyme that catalyzes the hydration and hydrolysis of methylthioacryloyl-CoA. *PloS. One*. 8:e63870. doi: 10.1371/journal.pone.0063870.

Todd, J. D., Curson, A. R. J., Dupont, C. L., Nicholson, P., and Johnston, A. W. B. (2009). The *dddP* gene, encoding a novel enzyme that converts dimethylsulfoniopropionate into dimethyl sulfide, is widespread in ocean metagenomes and marine bacteria and also occurs in some Ascomycete fungi. *Environ. Microbiol.* 11, 1376-1385. doi: 10.1111/j.1462-2920.2009.01864.x.

Todd, J. D., Curson, A. R., Kirkwood, M., Sullivan, M. J., Green, R. T., and Johnston, A. W. (2011). DddQ, a novel, cupin-containing, dimethylsulfoniopropionate lyase in marine roseobacters and in uncultured marine bacteria. *Environ. Microbiol.* 13, 427-438. doi: 10.1111/j.1462-2920.2010.02348.x.

Todd, J. D., Curson, A. R., Nikolaidou-Katsaraidou, N., Brearley, C. A., Watmough, N. J., Chan, Y., et al. (2010). Molecular dissection of bacterial acrylate catabolism - unexpected links with dimethylsulfoniopropionate catabolism and dimethyl sulfide production. *Environ. Microbiol.* 12, 327–343. doi:10.1111/j.1462-2920.2009.02071.x.

Todd, J. D., Kirkwood, M., Newton-Payne, S., and Johnston, A. W. (2012). DddW, a third DMSP lyase in a model Roseobacter marine bacterium, *Ruegeria pomeroyi* DSS-3. *ISME J.* 6, 223-226. doi:10.1038/ismej.2011.79.

Todd, J. D., Rogers, R., Li, Y. G., Wexler, M., Bond, P. L., and Sun, L.(2007). Structural and regulatory genes required to make the gas dimethyl sulfide in bacteria. *Science* 315, 666-669. doi: 10.1126/science.1135370.

Villar, E., Farrant, G. K., Follows, M., Garczarek, L., Speich, S., and Audic, S., et al. (2015). Environmental characteristics of Agulhas rings affect interocean plankton transport. *Science* 348:1261447.

Williams, B. T., Cowles, K., Bermejo Martínez, A., Curson, A. R., Zheng, Y., and Liu, J. (2019). Bacteria are important dimethylsulfoniopropionate producers in coastal sediments. *Nat. Microbiol*. doi:10.1038/s41564-019-0527-1.
